# In Heart Failure Patients with Left Bundle Branch Block Single Lead MultiSpot Left Ventricular Pacing Does Not Improve Acute Hemodynamic Response To Conventional Biventricular Pacing. A Multicenter Prospective, Interventional, Non-Randomized Study

**DOI:** 10.1371/journal.pone.0154024

**Published:** 2016-04-28

**Authors:** Maciej Sterliński, Adam Sokal, Radosław Lenarczyk, Frederic Van Heuverswyn, C. Aldo Rinaldi, Marc Vanderheyden, Vladimir Khalameizer, Darrel Francis, Joeri Heynens, Berthold Stegemann, Richard Cornelussen

**Affiliations:** 1 The Second Department of Coronary Artery Disease, Institute of Cardiology, Warsaw, Poland; 2 Department of Cardiology,Congenital Heart Diseases and Electrotherapy Silesian Center of Heart Disease, Zabrze, Poland; 3 Heart Center, Ghent University Hospital, Ghent, Belgium; 4 Guys and St Thomas NHS Trust, St Thomas Hospital, London, England; 5 Onze-Lieve-Vrouwziekenhuis Aalst, Aalst,Belgium; 6 Cardiac Electrophysiology Unit, Barzilai Medical Center, Ashkelon, Israel; 7 Faculty of Medicine, Imperial College Healthcare NHS Trust, London, England; 8 Bakken Research Center, Medtronic, Maastricht, The Netherlands; Kurume University School of Medicine, JAPAN

## Abstract

**Introduction:**

Recent efforts to increase CRT response by multiSPOT pacing (MSP) from multiple bipols on the same left ventricular lead are still inconclusive.

**Aim:**

The Left Ventricular (LV) MultiSPOTpacing for CRT (iSPOT) study compared the acute hemodynamic response of MSP pacing by using 3 electrodes on a quadripolar lead compared with conventional biventricular pacing (BiV).

**Methods:**

Patients with left bundle branch block (LBBB) underwent an acute hemodynamic study to determine the %change in LV+dP/dtmax from baseline atrial pacing compared to the following configurations: BiV pacing with the LV lead in a one of lateral veins, while pacing from the distal, mid, or proximal electrode and all 3 electrodes together (i.e. MSP). All measurements were repeated 4 times at 5 different atrioventricular delays. We also measured QRS-width and individual Q-LV durations.

**Results:**

Protocol was completed in 24 patients, all with LBBB (QRS width 171±20 ms) and 58% ischemic aetiology. The percentage change in LV+dP/dtmax for MSP pacing was 31.0±3.3% (Mean±SE), which was not significantly superior to any BiV pacing configuration: 28.9±3.2% (LV-distal), 28.3±2.7% (LV-mid), and 29.5±3.0% (LV-prox), respectively. Correlation between LV+dP/dtmax and either QRS-width or Q-LV ratio was poor.

**Conclusions:**

In patients with LBBB MultiSPOT LV pacing demonstrated comparable improvement in contractility to best conventional BiV pacing. Optimization of atrioventricular delay is important for the best performance for both BiV and MultiSPOT pacing configurations.

**Trial Registration:**

ClinicalTrials.gov NTC01883141

## Introduction

Cardiac Resynchronization Therapy (CRT) is an effective treatment for patients with systolic heart failure and a wide QRS, however approximately 30% of patients fail to respond.[1] Several mechanisms may explain this observation, including the presence of substrate not amenable to CRT, the absence of dyssynchrony, or the inability to achieve optimal resynchronization via the implanted system. Non-optimal left ventricular (LV) lead positioning has been proposed as a non-response factor[2, 3] and therefore pacing from multiple LV sites via a second LV lead[4, 5] or by multiple-electrodes pacing using a multipolar lead has been proposed to increase the response.[6–8] The use of multiSPOT pacing (MSP) via a quadripolar lead to improve CRT-response has been investigated with differing results. Some studies have failed to show additional benefit[9, 10] while others have shown small acute benefits.[11–15] These latter studies are difficult to interpret because of differing methodology including non-optimal atrio-ventricular (AV) delays.[11–15] Assessment of the true acute hemodynamic and clinical benefit of MSP requires assessment in a robust and reliable fashion to allow conclusions can be drawn with an acceptable degree of certainty before this treatment can be adopted into clinical practice.

### Aim

The iSPOT study intended to assess the positive LV +dP/dt max achieved by multiple (3) LV pacing SPOTs, i.e. MultiSPOT configuration in comparison to the response achieved by the current standard BiV pacing configuration. The protocol included a rigorous methodology to decrease measurement variability and to be patient-tailored (through individualized AV-delay) in order to provide a justified conclusion to the acute hemodynamic benefit of MSP [16]. In addition, only left bundle branch (LBBB) patients were selected in order to adequately address the potential benefit of MSP in this large CRT patient population. In addition to the hemodynamic evaluation, electrical parameters including Q-LV timing and QRS-width for all the pacing configurations were measured and compared.

## Material and Methods

### Study design

The study was an international, prospective, interventional, non-randomized research study conducted at 7 high-volume sites. Inclusion and exclusion criteria are listed in Table 1. In eligible patients, contractility was measured using LV+dP/dt max at various LV pacing sites. The hemodynamic study was done immediately prior to a planned CRT-implant either concomitant or later. All patients were enrolled prospectively, served as their own control and gave written informed consent before the start of the study. The study was approved by government authorities and the local ethical committees. All ethics committees approvals are listed in Table 2.

**Table 1:** Study inclusion and exclusion criteria.

Inclusion criteria	Exclusion criteria
Sinus rhythm	Permanent atrial fibrillation/ flutter or tachycardia
Left bundle branch block	Recent myocardial infarction, within 40 days prior to enrollment.
Left ventricular ejection fraction ≤ 35%	Coronary artery bypass graft or valve surgery, within 90 days prior to enrollment
Heart failure in II- ambulatory IV NYHA class	Heart transplantation, or patient is actively listed on the transplantation list
Written informed consent	Left ventricular assist device
Optimal heart failure pharmacotherapy for at least 3 months	Severe renal disease (up to physicians discretion)
	Continuous or uninterrupted infusion (inotropic) therapy for heart failure (≥ 2 stable infusions per week
	Severe aortic stenosis (with a valve area of <1.0 cm^2^ or significant valve disease expected to be operated within study period)
	Complex and uncorrected congenital heart disease
	Mechanical heart valve
	Pregnant or breastfeeding women, or women of child bearing potential and who are not on a reliable form of birth control
	Enrollment in at least one concurrent study

**Table 2:** Numbers of local and central ethics committees approvals for all participating sites.

Site	Local ethics committee approval number	Central ethics committee approval (CA) number	CA Agency
Belgium—Aalst	B670201215037	80M0524	Federal Agency for Medicines and Health Products
Belgium—Ghent	B670201215037	80M0524	Federal Agency for Medicines and Health Products
Israel—Ashkelon	0092-12-BRZ	HTA6661	Ministry of Health
Poland—Warsaw	KNW/0022/KB/W/9/13	No CA needed	No CA needed
Poland—Katowice	KNW/0022/KB1/26/IV/13	No CA needed	No CA needed
UK—London (Imperial College Healthcare NHS Trust	12/LO/1762	CI/2013/0013	Medicines & Healthcare Products Regulatory Agency
UK—London (St Thomas Hospital)	12/LO/1762	CI/2013/0013	Medicines & Healthcare Products Regulatory Agency

Following a coronary venogram to identify the target vessels for LV stimulation a quadripolar lead (*Performa*^*TM*^, *Medtronic plc*, *Minneapolis*, *MN or Quartet*^*TM*^, *St Jude Medical*, *Sylmar*, *CA*) or a decapolar catheter (*TORQR*, *Medtronic plc*, *MSP*, *MN*) was advanced to the target vein by subclavian approach: the LV electrode positions were determined using a qualitative approach; using the MRI images to determine the length of the LV axis (AV-plane of the coronary sinus vein (designated base of the heart), to the apex) and the fluoroscopy images to qualitatively determine the distance of the electrodes from the base. CRT contractility was measured using LV+dP/dt via a *Micro-Cath*^*™*^ *catheter Millar*,*Texas*, *USA* placed in the left ventricle via the femoral artery.

#### Pacing system

Four individual cardiac stimulators (*5388 DDD*, *Medtronic plc*) synchronized by a *Model 2090* programmer were used for simultaneous, independent, multi-site pacing of the left ventricle. To facilitate connections and improve safety during the EP procedure, a custom-made connection box was used for simultaneous stimulation at various sites.

#### Recording equipment

Throughout the procedure all physiological signals (intra-cardiac EGM, ECG, and left ventricular pressure) were acquired real time with a 32 channel physiological recording system (*Porti*, *TMSI*, *Twente*, *NL*) and recorded using customized software. All configurations and experimental settings were annotated digitally on the same system. Signals were visualized on the laptop computer throughout the study to assess signal quality and to confirm adequate therapy delivery. No data analysis was performed at this stage.

### Study protocol

The following LV pacing configurations were evaluated:

1. Biventricular pacing with pacing RV and LV on the distal electrode (LV-dist)
2. Biventricular pacing with pacing RV and LV on the mid electrode (LV-mid)
3. Biventricular pacing with pacing RV and LV on the proximal electrode (LV-prox)
4. RV and simultaneous LV pacing on all three electrodes (MSP)

To avoid the effects exerted on LV performance by different heart rates, pacemakers were programmed to pace in DDD mode at a lower rate of 100 bpm to ensure atrial and ventricular capture. It was decided to have one fixed heart rate for all patients and 100 bpm was well tolerated. In addition, the higher heart rate was found to be more sensitive to changes in contractility when using the different CRT configurations. For all configurations the VV delay was set at 0 ms. For each configuration, measurements were performed at 5 different AV delays: the patient specific optimal AV delay determined by using the *CardioSync*^*TM*^ algorithm, the optimal AV delay +/-20 and +/-40 ms [17]. All measurements were repeated four times for each pacing configuration and AV-delay to increase the signal to noise ratio. Each setting lasted 20 beats (10–15 s) interspersed with baseline AAI pacing.

### Data Analysis

Data analysis was reviewed off-line and where needed the correct start and end of the AAI and DDD pacing sequences were manually corrected. Premature complexes, and beats with loss of capture were manually annotated. Thereafter, final analysis was done on annotated data files using a validated custom-made program written in *Matlab*^*™*^.

The paced QRS duration was measured from the beginning of the ventricular pacing spike to the end of the QRS complex in surface ECG. The Q-LV interval was defined as the interval from the onset of the intrinsic QRS on the surface ECG to the first large positive or negative peak of the LV electrogram. Q-LV-timing data are expressed as Q-LV-ratio which is the ratio of the Q-LV-time and QRS-width.

#### Statistical analysis

Statistical analysis was performed using SAS 9.4 (SAS Institute,Cary NC) and R (versions up to 3.2.0). The primary objective was to compare the acute hemodynamic response of a MultiSPOT LV pacing configuration to a BiV configuration using %-change LV+dP/dtmax from baseline (AAI pacing). In the first step, the maximal average—change LV+dP/dtmax response was calculated for each subject and each configuration by a regression analysis constructing a quadratic curve through all AV-delays. From this step, a %-change of LV+dP/dtmax value for each patient within each configuration was obtained. In the second step, paired t-tests were performed to compare the configurations to each other. A subgroup analysis was performed comparing MultiSPOT and BiV configurations within ischemic and non-ischemic patients. Superiority testing at a 0.05 significance level was performed after significance of non-inferiority at a 0.025 level and 4% non-inferiority margin. Since non-inferiority testing was significant in most comparisons, only p-values for superiority are presented (unless indicated otherwise). The hypotheses are explained below:

The null hypothesis for non-inferiority was as follows:

$${\text{H}}_{0}:\;\Delta \text{multispot }-\;\Delta \text{ BiV }\leq \;-4\%\;\left(\text{inferiority}\right),$$

where Δ_multispot_ indicates the percentage change LV dP/dt max for the multispot pacing configuration and Δ_BiV_ indicates the percentage change for a given BiV pacing configuration.

This null hypothesis was tested against the alternative hypothesis:

$${\text{H}}_{1}:\;\Delta \text{multispot }-\;\Delta \text{ BiV }>\;-4\%\;\left(\text{non-inferiority}\right).$$

A p-value smaller than 0.025 was considered significant, and consequently the null hypothesis was rejected. Testing at 0.025 level means that p-values smaller than 0.025 are considered significant. The non-inferiority margin of 4% was chosen based on literature research. The non-inferiority margin is here 4% and represents the acceptable difference between multispot and BiV pacing above which we would declare multispot to be non-inferior.

After rejection of non-inferiority of multispot to BiV pacing, superiority of multispot pacing to BiV pacing was tested. The following null hypothesis was considered:

$${\text{H}}_{0}:\;\Delta \text{multispot }-\;\Delta \text{ BiV }=\;0\%$$

versus the alternative hypothesis

$${\text{H}}_{1}:\;\Delta \text{multispot }-\;\Delta \text{ BiV }\neq \;0\%.$$

This analysis was pre-specified in the statistical analysis plan of the iSPOT study, and is in line with standard statistical analysis for non-inferiority trials.

No correction for multiple testing was performed. To determine the correlations between absolute LV+dP/dtmax value and Q-LV or QRS duration as well as the correlations between % change LV+dP/dtmax value and Q-LV ratio or QRS ratio, linear multiple regression analysis was used. Data are expressed as mean±SD (unless indicated otherwise).

## Results

### Baseline Characteristics and Hemodynamic Results

**Fig 1:**
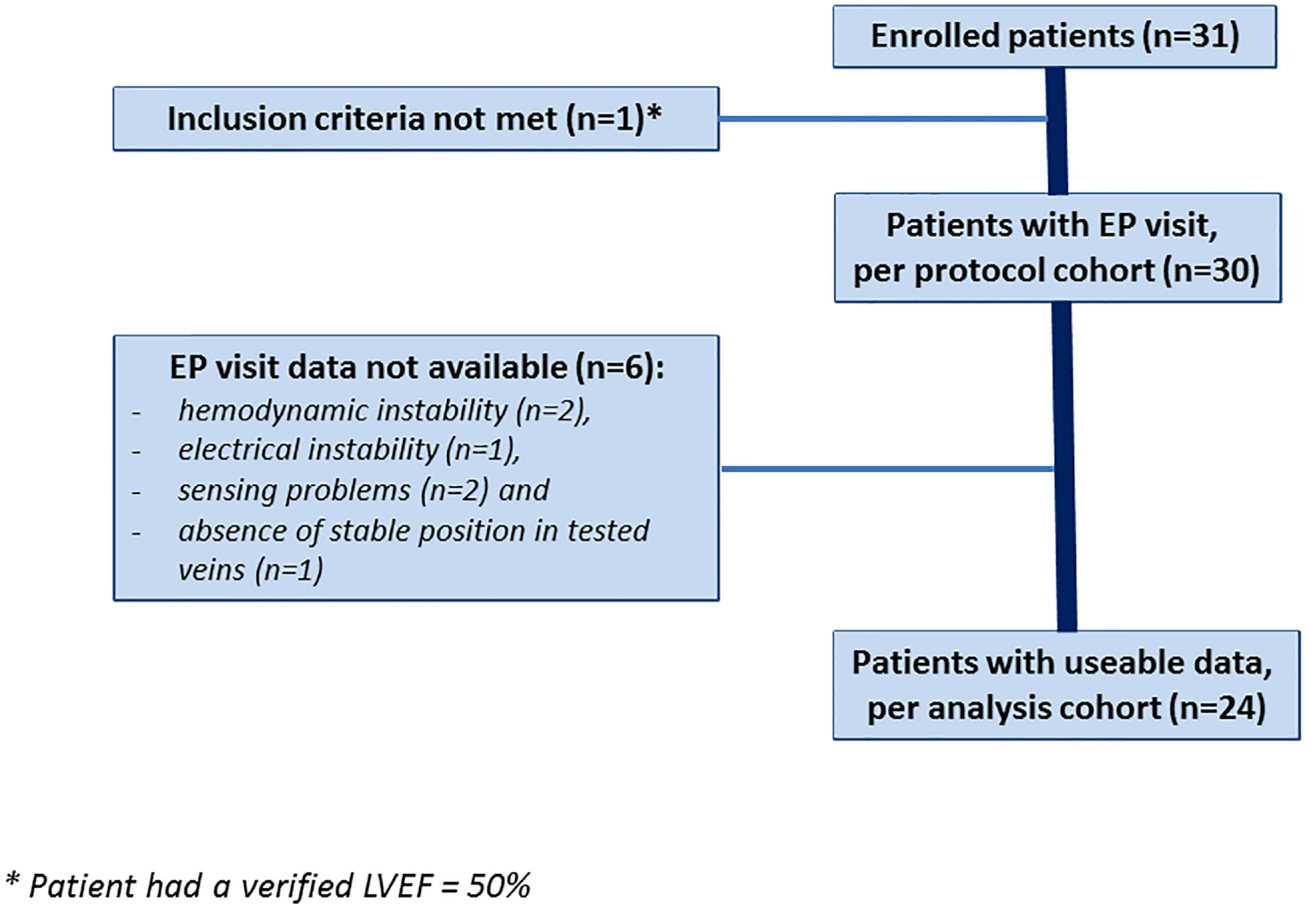
Flowchart of the enrolled patients.

Full protocol was obtained in 24 subjects (77%) from the 31 patients enrolled in the iSPOT study. One subject was excluded from the cohort for not fulfilling all the inclusion criteria. Fig 1 shows a flowchart of the enrolled patients and the characteristics of 30 patients are shown in Table 3. The excluded patients had hemodynamic instability (n = 2), electrical instability (n = 1), sensing problems (n = 2) and absence of stable position in tested veins (n = 1). Ischemic aetiology was observed in 58% of patients. Tested LV lead locations were: a) posterolateral in 12 patients (50%), b) lateral in 11 (46%) and c) anterolateral in one patient (4%). Bipolar RV stimulation was performed from either RV-apex in 16 pts (63%) or RV-septum in 8 pts (37%). Anatomic evaluation indicated the LV distal tip was placed on average 58% from the base indicating that the distal electrode was placed more in the mid instead of the true apical region. Baseline LV+dP/dtmax measured with AAI pacing mode was 677±215 mmHg/sec and increased (taking into account all AV-delays tested) in the LV-distal, LV-mid, LV-prox, and multiSPOT configuration to 818±217, 822±248, 819±226 and 845±235 mmHg/sec respectively.

**Table 3:** Characteristics of studied population. QRS morphology was classified according to AHA/ACCF/HRS guidelines. Patient aetiology other means inflammation induced cardiomyopathy.

Patient Characteristics	Total Subjects (N = 30)
**Gender (N,%)**	
Male	24 (80.0%)
Female	6 (20.0%)
Age (years)	61.0 ± 13.0
Heart Rate (bpm)	74.4 ± 14.7
Systolic Blood Pressure (mmHg)	126.4 ± 19.5
Diastolic Blood Pressure (mmHg)	77.0 ± 11.6
LV Ejection Fraction (%)	24.3 ± 6.1
**New York Heart Association (N,%)**	
Class II	11 (36.7%)
Class III	19 (63.3%)
**Cardiomyopathy**	
Dilated/congestive	14 (46.7%)
Ischemic	17 (56.7%)
Other	1 (3.3%)
QRS duration (ms)	170.9 ± 20.0
LBBB (N,%)	30 (100%)

#### Hemodynamic Response after AV delay optimisation

**Fig 2:**
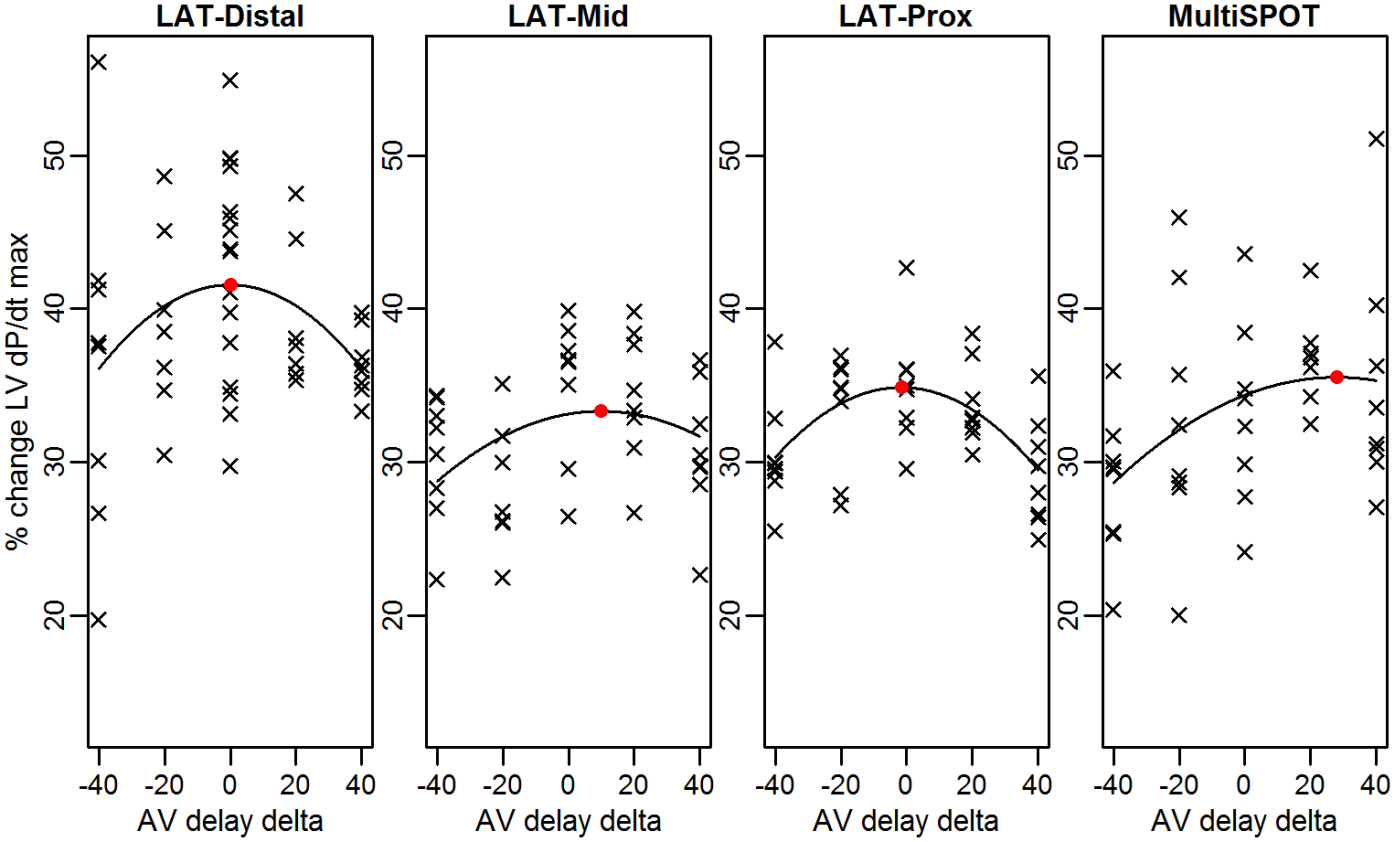
Regression curve constructed through percentage LV+dP/dtmax changes during each configuration depending on AV delay in one patient. Red dot indicates calculated maximal average response at AV-best. AV = 0 indicates AV-delay calculated by CardioSyncTM formula. AV-delay delta stands for change from AV-delay calculated by CardioSyncTM formula in ms.

**Fig 3:**
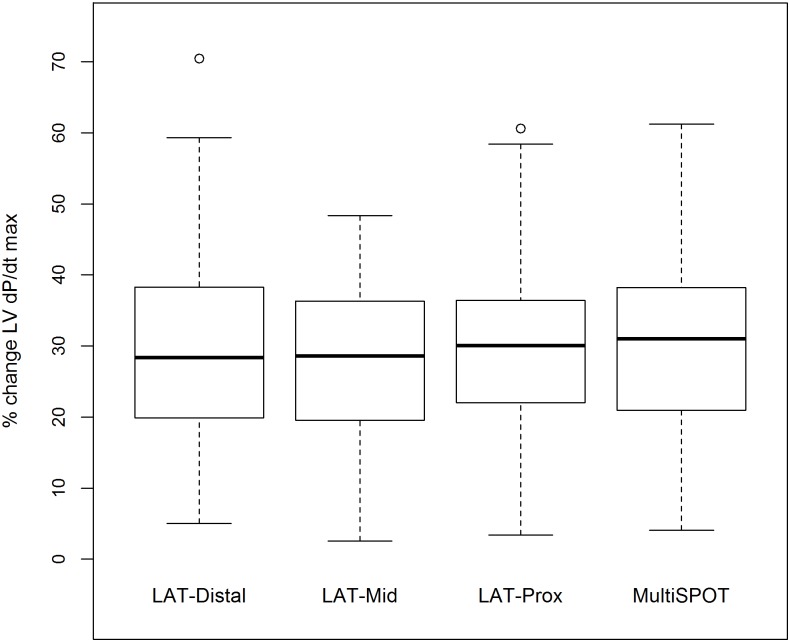
Boxplot of LV+dP/dtmax % increase for the different configurations. Distal, mid and prox denote LV-electrode position on the LV-lead. MultiSPOT denote pacing on all three LV-electrode positions simultaneously. Solid line depicts mean value and boxes are 75 and 25 percentiles. Whiskers represent 2.5 and 97.5% percentiles.

AV delay curves for all configurations and subsequent determination of AV delays with the maximal LV+dP/dtmax for 1 patient is depicted on Fig 2. Following AV delay optimisation, LV+dP/dtmax increased respectively by 28.9±3.2% (LV-dist) (mean±standard error), 28.3±2.7% (LV-mid), 29.5±3.0% (LV-prox) and 31.0±3.3% (MultiSPOT) as compared to baseline. Individual response measured by LV+dP/dtmax is shown on Fig 3. No difference in LV+dP/dtmax was noted between the different configurations for the best AV interval (max difference of 2.8±1.5% between MultiSPOT and BiV mid, all p-values >0.05). The difference between the optimal AV-delay calculated by the *CardioSync*^*TM*^ Algorithm (depicted as AV = 0 on Fig 2) and the real optimal AV-delay, which lead to maximal hemodynamic response as determined by regression analysis, ranged from -1.7ms (LV-prox) to +5.8ms (LV-mid). The optimal AV-interval, calculated by the CardioSync^™^ was 164±31ms, ranging from 110 to 240ms. The PQ-time during baseline AAI-pacing was 257±55ms, ranging from 180 to 387ms.

#### Hemodynamic Response and Aetiology

MultiSPOT pacing revealed a significantly higher % increase in LV+dP/dtmax response than BiV dist pacing in ischemic patients (mean difference ± standard error: 5.7 ± 2.2, superiority testing: p = 0.0224) but MultiSPOT was not significantly different to BiV distal pacing in non-ischemic patients (mean difference ± standard error: -2.4±1.7, inferiority testing: p = 0.1883). No significant differences in LV+dP/dt were noted when comparing MSP versus BiV mid or MSP versus BIV prox in both ischemic and non-ischemic patients (p > 0.083).

#### Individual Response

**Fig 4:**
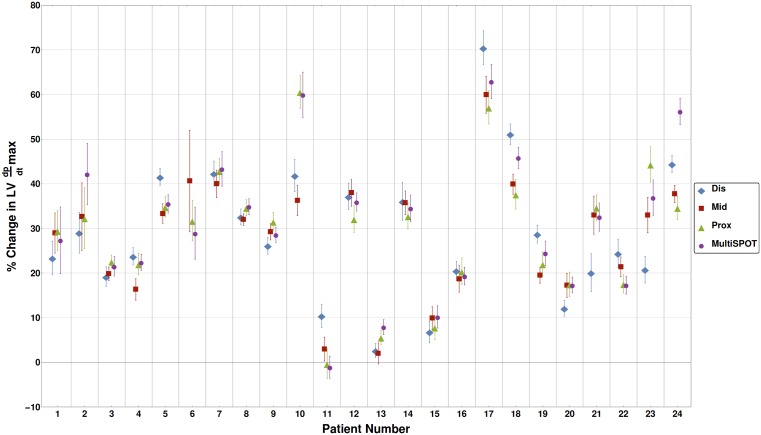
Individual contractility data per patient. Presented are the maximal % LV+dP/dtmax values (mean and confidence intervals) for all 24 patients for all different BiV and MultiSPOT configurations. Patient 6 has no LV-distal data because of intolerable phrenic nerve stimulation at this position. For more explanation see text.

Patients were categorized according to their max response in LV +dP/dtmax (Fig 3). It was noted that MSP performed better (defined as an increase in LV +dP/dt max of ≥ 10% over best BiV) in 2 patients (Fig 4; patients 2 and 24). MSP was not effective in increasing LV+dP/dtmax values in acute non- or low responders (<10% increase in LV+dP/dtmax values). In two of 24 patients one conventional BiV setting resulted in >10% better hemodynamic response compared to MSP suggesting a possible detrimental effect of MSP in these patients.

### Impact of configuration upon QRS-width changes

**Fig 5:**
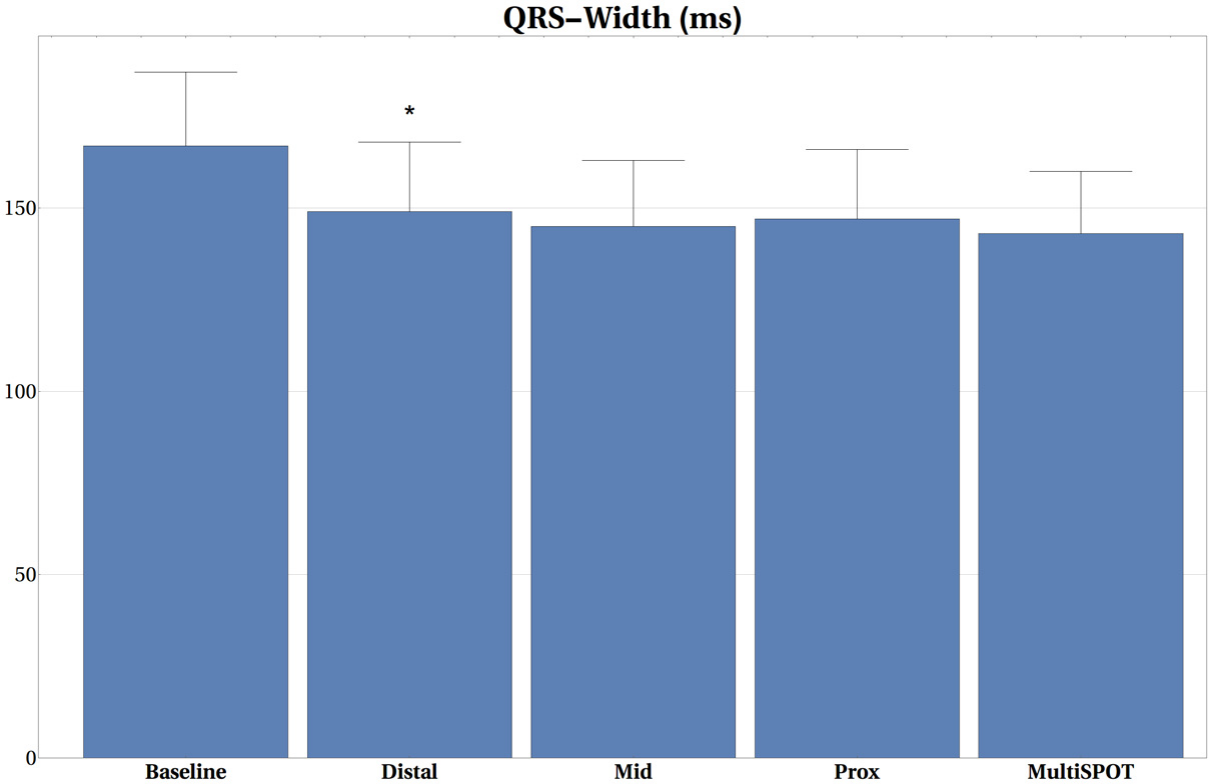
QRS width values at different pacing modes. QRS-width reduction by BiV pacing approximates 15% and is only slightly more reduced by MultiSPOT pacing. It has weak correlation with % LV+dP/dtmax increase (R = -0.136). (B)

**Fig 6:**
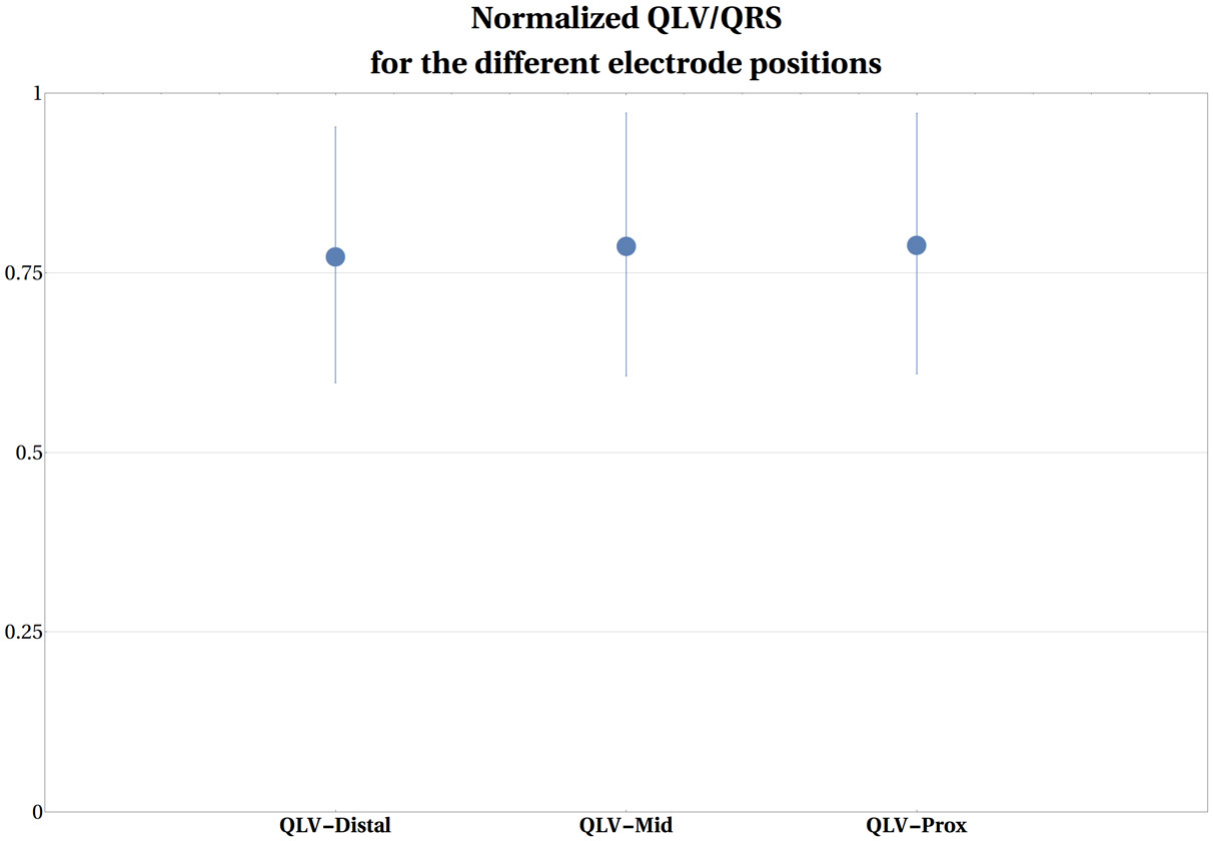
QLV/QRS values upon different pacing modes. Normalized QLV/QRS values for the different electrodes on the Multi-electrode LV lead.Data and correlations are presented as mean±SD. * indicates significant different from MultiSPOT.

Conventional BiV pacing decreased the QRS-width from 167±19ms to 149±19ms, 145±19ms, 147±19ms during LV-distal, LV-mid and LV-prox pacing, respectively. There was a small additional decrease in QRS width during MSP pacing to 143±17ms, which was significant in comparison to BiV-dist (p = 0.004) but to none of the other configurations (BiV-mid; p = 0.121 or BiV-prox; p = 0.056). No strong correlations between absolute LV+dP/dtmax and QRS-width (R = -0.310) or % change LV+dP/dtmax and QRS-width ratio (i.e. QRS-pacing setting/QRS-baseline) were noted (R = -0.136). Figs 5 and 6 summarize QRS and the normalized Q-LV-values for the different pacing configurations. The normalized QLV timings were similar for all the three electrodes on the LV-lead, occurring at 0.782±0.077, 0.795±0.038 and 0.797±0.038 QRS duration for distal, mid and prox electrode positions, respectively. This indicates that LV activation occurs late, that is at 80% of the QRS-time. The average inter-electrode time delay between earliest and latest activation was 13±8 ms, varying from 1 to 21 ms. No correlation was observed between Q-LV-timing and LV+dP/dtmax (R = 0.053) and between max %-change LV+dP/dtmax and Q-LV-ratio (i.e. Q-LV/QRS-width) (R = 0.007).

## Discussion

The present study was designed to investigate the feasibility of increased efficacy of multipoint pacing in CRT-eligible patients, using invasive measurements only in experienced hospitals to execute safely this type of research (see: section *limitations*). The study also included less invasive measurements (eg electrical parameters like Q-LV and QRS-width) which are linked to the acute possible beneficial effect of multipoint pacing. The study demonstrated that after AV delay optimization MSP had no additional acute improvement in systolic LV pump function compared to conventional biventricular pacing in LBBB patients with heart failure. In this patient cohort the optimal hemodynamic response could be achieved by tailored AV-delay optimization using the *CardioSync*^*TM*^ algorithm.

### Hemodynamic effects of conventional biventricular pacing

The differences in hemodynamic benefit of BiV pacing compared to AAI) using the different electrodes on the multipolar lead were small (distal 28.94%, mid 28.26%, and proximal electrode 29.45%) but the absolute value relatively high when compared to other studies. The high acute response to CRT in the current study is likely explained by the optimal positioning of the LV lead in the lateral vein [3, 18, 19] and the specific pacing protocol in which we measured the hemodynamic response by repetitive measurements of 10–20 seconds which differs from the more steady state measurements over a minute pacing interval generally used. In addition, only patients with LBBB (known high responders) were included. Moreover, a relatively high pacing rate was used together with individual optimization of AV delays, maximizing the CRT-effect.

### Hemodynamic effects of MultiSPOT pacing and comparison with previous studies

The findings of the current study do not support the hypothesis that simultaneous pacing using all three electrodes (MultiSPOT; MSP) increased the acute hemodynamic CRT response through activation of a greater amount of LV tissue in patients with LBBB beyond conventional CRT. For comparison of our results to published data, we can classify prior studies of multisite pacing into those using simultaneous stimulation with a VV-delay of 0 ms (simultaneous MSP) and those using varying delays between the LV electrodes (sequential MSP). In the simultaneous MSP studies, the acute hemodynamic benefit of MSP was either small and non-significant[10, 20] or small but statistically different[12, 15] compared to conventional BiV-pacing. The difference may be explained by the AV-delay as in the studies which showed positive hemodynamic effect of MSP[12, 15] the AV-delay was similar for all patients[15] while in the studies which did not show significant benefit an individualized patient-optimal AV-delay was used.[10, 21] This is in-line with the current findings, where AV-optimization would suggest that MSP has no incremental benefit and, a previous study using two LV-leads.[21] Although there is a lot of debate what the exact AV-delay for the individual patient should be, it is well recognized that not every patient benefits from a standardly chosen AV-delay.[17] In some “sequential MSP-studies” to ensure full LV capture the AV-delay was kept very short (25–50 ms)[13, 22] whereas others used a more physiological AV-delay.[9, 14] In these studies MSP demonstrated a small and variable acute hemodynamic benefit.[23] In addition, one previous study has shown that MSP improves longer-term CRT response.[24] Interpretation of the results of these studies might be hampered by the methodologies used in these papers which are not always comprehensive and AV-optimization was not performed. The rationale for using sequential LV-pacing is unclear. The electrical distance between the electrodes of current quadripolar leads measured in the normal CRT population during sinus rhythm ranges between 10 and 20ms (current study and[10]). This may limit possible intervention with an electrical therapy like CRT. Ideal inter-electrode spacing (maximal anatomic distance on quadripolar lead) to achieve maximal MSP response needs to be investigated.[25]

### Individual patient response to MultiSPOT pacing

In our study it was noted that MSP performed better (defined as an increase in LV+dP/dt max of more than 10% over best BiV) in 2 patients. Previous studies have indicated that MSP could be more beneficial in ischemic patients[11, 26] and both our ‘MSP responders’ had an ischemic aetiology. Studies have suggested that a non-responder could be turned into a responder with MSP[27] however, if we define non-responders in our study as patients with ≤10% increase in LV+dP/dtmax in any BiV configuration, MSP could not turn these patients into a responder (i.e.>10% increase in LV+dP/dtmax). It should also be noted in the current study that MSP could have detrimental effects on hemodynamic function when compared to the best BiV pacing configuration with 2 out of 24 (8.3%) patients with one BiV setting more than 10% better than MSP which has also been shown previously.[12] The nature of this phenomenon is unclear but the creation of functional block, interference with the sequence of activation or mitral valve function might be implicated. Such findings should raise caution in the use of MSP in CRT[10] and more mechanistic studies are warranted to investigate the role of MSP in individual patients. In line with this Sohal et al[20] recently showed that a significant hemodynamic benefit of MSP was observed only in hemodynamic non-responders to conventional BiV pacing and that the positive effect of MSP was only seen in patients that did not fulfill strict criteria for LBBB and that tended to have an ischemic aetiology. In keeping with the findings of Sohal et al a comparison of ischemic patients versus non-ischemic patients in the current study revealed that in ischemic patients, MSP increased LV+dP/dtmax when compared to LV-distal pacing but not to LV-mid or LV-prox pacing. These findings indicate that in this LBBB-specific population lead location within the coronary vein is important.

### MultiSPOT pacing and its effects on electrical parameters

Intuitively a more simultaneous activation of the LV with MSP would be expected to enhance resynchronization and thereby result in hemodynamic benefit. Data on QRS-narrowing as an indicator for CRT-response are controversial.[2, 28, 29] lt was found that compared to conventional BiV-pacing MSP elicited a significantly greater (absolute difference = 4ms) QRS-width narrowing.[15] In the current study MSP resulted in a significant QRS-narrowing only when compared to conventional BiV pacing from the distal electrode.[15] Importantly the correlation between QRS-width and contractility increase was weak in our study, indicating that QRS-change with pacing may not be a reliable predictor of acute hemodynamic response. Other modalities such as vectorcardiography might be a more promising electrophysiological predictor of CRT response[30], perhaps in conjunction with known predictors such as baseline contractility (LV+dP/dtmax) value, QRS duration or morphology.[18] In keeping with our findings LV activation times measured by invasive mapping in a canine model showed that MSP with up to seven stimulation sites is capable of decreasing total LV-activation time but the improvement in contractility was non-significant when compared to the optimal single BiV stimulation.[3] Retrospective analyses show that patients with LV leads implanted at sites with a long Q-LV are more likely to respond to CRT[31] and Zanon et al[15] found a good correlation between Q-LV and hemodynamic response with MSP. Our study did not confirm this finding but this is likely due to the comparable Q-LV-timings because of using the lateral vein in all patients.

### Limitations

The present study used the change in invasively measured left ventricular +dP/dtmax as parameter to define acute CRT response, while other hemodynamic parameters can be used such as stroke work using pressure-volume loops or blood pressure for acute CRT-feedback [32]. An acute increase in one of these parameters identifies patients who are most likely to respond to CRT. But it is important to realize that these observations do not provide an insight into which invasive parameter is superior for identifying long-term response to CRT and how they relate to the traditional identifiers of response. This study does not provide an answer to this question either, but with the specific repetitive methodology used the likelihood of acute parameters being predictive should increase considerably. [33] In this study only patients with broad LBBB morphology were deliberately included. Therefore, the results may not be valid for other patients groups such as ischemic patients or patients without strict LBBB where CRT response is reduced. The number of patients (although from multiple centers) was small but comparable with previous (single-center) studies. The intention was to focus on AV-delay. Due to time constraint we only tested MSP in one vein and did not optimize VV-delay. Protocol of the study was limited by ethical considerations not to exceed “skin-to-skin” procedure time, compared to an accepted average time of the procedure in less experienced centers. The data from the present study need to be confirmed in larger prospective studies delineating different CRT-populations. One must be aware that management of heart failure requires a multimodal approach. Besides the optimization of device settings, also lifestyle modifications and sustained optimized medication play very important roles.

## Conclusions

In this heart failure population with wide left bundle branch block and indication for cardiac resynchronization therapy, tested in a noise-minimizing high-replicate protocol with optimized atrio-ventricular delay, MultiSPOT left ventricular pacing demonstrated no significant acute improvement in contractility over the one best biventricular pacing site. Future studies to assess the long term effect and possible multisite pacing benefits in patients with ischemic aetiology and other forms of intraventricular conduction disorders are warranted. Optimization of atrioventricular delay affected the best performance of all pacing configurations.

## Supporting Information

S1 Protocol Left Ventricular MultiSpot Pacing for CRT (iSpot) Clinical Investigational Plan. (PDF)

S1 TREND Checklist (PDF)
