# Supplementary material for: In Heart Failure Patients with Left Bundle Branch Block Single Lead MultiSpot Left Ventricular Pacing Does Not Improve Acute Hemodynamic Response To Conventional Biventricular Pacing. A Multicenter Prospective, Interventional, Non-Randomized Study
Source: PLoS One. 2016 Apr 28;11(4):e0154024. doi: 10.1371/journal.pone.0154024 (PMC4849737; doi:10.1371/journal.pone.0154024)
Supplement: S1 Protocol — (PDF) [file pone.0154024.s001.pdf]

Left Ventricular MultiSpot Pacing for CRT (iSpot)

Clinical Investigational Plan

VERSION 3

24 February 2014

Bakken Research Center BV, Medtronic
Endepolsdomein 5, 6229 GW
Maastricht, The Netherlands

Table of Contents

1 ADMINISTRATIVE INFORMATION 4

1.1 SPONSOR INFORMATION 4
1.2 PARTICIPATING INVESTIGATORS AND IRBS/MECS 4

2 STUDY SUMMARY 5

3 STUDY BACKGROUND AND JUSTIFICATION 7

4 SYSTEM DESCRIPTION AND INTENDED USE 9

4.1 PACING DEVICES 9
4.2 LEADS AND ELECTROPHYSIOLOGICAL CATHETERS 10
4.3 ACQUISITION SYSTEM 11

5 REGULATORY COMPLIANCE 15

6 METHODOLOGY 15

6.1 STUDY DESIGN 15
6.2 STUDY OBJECTIVES 15
6.2.1 Primary Objective 15
6.2.2 Secondary Objectives 15
6.3 SUBJECT SELECTION 16
6.3.1 Inclusion Criteria 16
6.3.2 Exclusion Criteria 16
6.4 MINIMIZATION OF BIAS 17
6.5 DATA MONITORING COMMITTEE 17

7 STUDY VISITS AND PROCEDURES 18

7.1 CENTER/INVESTIGATOR SELECTION CRITERIA 18
7.2 CENTER ACTIVATION 18
7.2.1 Equipment Requirements 19
7.3 PATIENT INFORMED CONSENT PROCESS 19
7.4 STUDY VISITS 21
7.4.1 Baseline visit 21
7.4.1.1 Magnetic Resonance Imaging (MRI) 22
7.4.2 Electrophysiological (EP) study visit 22
7.5 SUBJECT EXIT AND WITHDRAWAL 26
7.5.1 Subject Initiated Withdrawal 27
7.5.2 Investigator Initiated Subject Withdrawal 27
7.6 OVERVIEW OF DATA COLLECTION REQUIREMENTS 27

8 STATISTICAL METHOD AND DATA ANALYSIS 28

8.1 SAMPLE SIZE JUSTIFICATION 28
8.2 STUDY ENDPOINT 29
8.3 ANALYSIS METHODS 29
8.4 ANALYSIS POPULATIONS 30
8.5 INTERIM ANALYSIS 30

9 DATA AND QUALITY MANAGEMENT 30

APPENDIX A: STUDY OVERVIEW 32

APPENDIX B: PATIENT INFORMED CONSENT SAMPLE 37

APPENDIX C: REFERENCES 44

LIST OF FIGURES

Figure 1: Figure depicting the different ventricular lead positions 7
Figure 2: iSPOT equipment set-up 10
Figure 3: Multispot electrophysiological catheters...11
Figure 4: Description of the pacing protocol..25

LIST OF TABLES

Table 1: Sponsor Contact Information 4
Table 2: Participating Investigators 4
Table 3: Devices and accessories..12
Table 4: Data Collection Requirements...28

1 Administrative Information

1.1 Sponsor Information

The sponsor of this study is Bakken Research Center BV, Medtronic Endepolsdomein 5, 6229 GW, Maastricht, The Netherlands. Table 1 lists the sponsor contact information. This information is subject to change during the course of the clinical study. Periodic updates to study contact information will be sent to the centers.

Table 1: Sponsor Contact Information

Role / Name	Phone	Fax	Email
Scientist/ Richard Cornelussen	+31 (43) 3566651	+31(43) 3566516	Richard.cornelussen@medtronic.com
Scientist/ Berthold Stegemann	+49 (288) 92894161	+31(43) 3566516	Berthold.stegemann@medtronic.com
Clinical Research Specialist/ Joeri Heynens	+31 (43) 3566713	+31(43) 3566516	Joeri.heynens@medtronic.com

1.2 Participating Investigators and IRBs/MECs

Table 2 lists the participating investigator/center details. Additional sites could be added during the study. A complete list of participating investigators and the IRB/MEC details will be distributed under a separate cover upon request.

Table 2: Participating Investigators

Investigator
**Prof. Dr. D Francis**<br>Cardiologist<br>Faculty of Medicine<br>Imperial College Healthcare NHS Trust<br>St Mary's Hospital<br>Praed Street<br>W2 1NY<br>London, UK
**Dr. A Rinaldi**

Cardiologist at Guys and St Thomas NHS Trust
St Thomas Hospital
Lambeth Palace Road
SE1 7EH
London, UK

Prof Dr. Z Kalarus
Head of Department of Cardiology, Congenital Heart Diseases and Electrotherapy,
Medical University of Silesia, Silesian Center for Heart Disease,
Ul. Szpitalna 2, 41-800
Zabrze, Poland.

Prof Dr. F van Heuverswyn
Cardiologist-Electrophysiologist
Hartcentrum 10K12
Universitair Ziekenhuis Gent
De Pintelaan 185
B-9000 Gent, Belgium

Prof Dr. M Vanderheyden
Cardiologist-Electrophysiologist
Campus Aalst
Onze-Lieve-Vrouwziekenhuis Aalst
Moorselbaan 164
9300 Aalst, Belgium

Dr. Khalameizer
Head of Cardiac Electrophysiology Unit
Barzilai Medical Center
2 Hahistadrut Street
Ashkelon 78278
Barzilai, Israel

Dr. Maciej Sterliński
Instytut Kardiologii
Klinika Choroby Wieńcowej
ul. Spartańska 1
02-637 Warszawa, Poland

Prof. Andrzej Przybylski
Instytut Kardiologii
Klinika Zaburzeń Rytmu Serca
ul. Alpejska 42
04-628 Warszawa, Poland

2 Study Summary

This clinical trial is an exploratory, prospective, interventional, non-randomized multi-center research study assessing augmentation of contractility using

positive left ventricular (LV) dP/dt max across LV pacing site(s) in patients indicated for cardiac resynchronization therapy (CRT).

During a research study, an electrophysiological (EP)-exploratory procedure or prior to CRT-implant, a venogram will be obtained to identify the target vessels for LV stimulation. Leads will be placed (temporarily) in three coronary veins; in the anterior, posterior and lateral branches. No more than two left ventricular leads will placed at the same time.

Three LV pacing configurations will be evaluated:
- Biventricular pacing (BiV:1 RV lead and 1LV lead),
- Tri-ventricular pacing (Multivein: 1 RV lead and 2 LV leads) or
- Quadri-ventricular pacing (MultiSpot: 1 RV lead 1 LV catheter with LV pacing spots)
  and the corresponding positive LV dP/dt max will be measured.

BiV pacing will be performed at each of the three coronary veins. The total number of BiV settings is 5 being:

- **RV – LV Lateral – Proximal (Reference: Standard CRT)**
- RV – LV Lateral – Mid
- RV – LV Lateral – Distal
- RV – LV Posterior
- RV – LV Anterior

Multivein-pacing will be performed in the posterior and anterior target vessels (in each vein one spot). There is only one multivein-setting, being:

- RV – LV Anterior – LV Posterior

Multispot-pacing will be performed using an EP catheter at the “normal” LV lead implant site (i.e., (postero)-lateral target vessel). There is only one Multispot-setting tested, being:

- RV – LV Lateral Proximal – LV Lateral Mid – LV Lateral Distal (Main Assessment)

Within this study the total number of settings is 7, using three lead configurations (see also Figure 1).

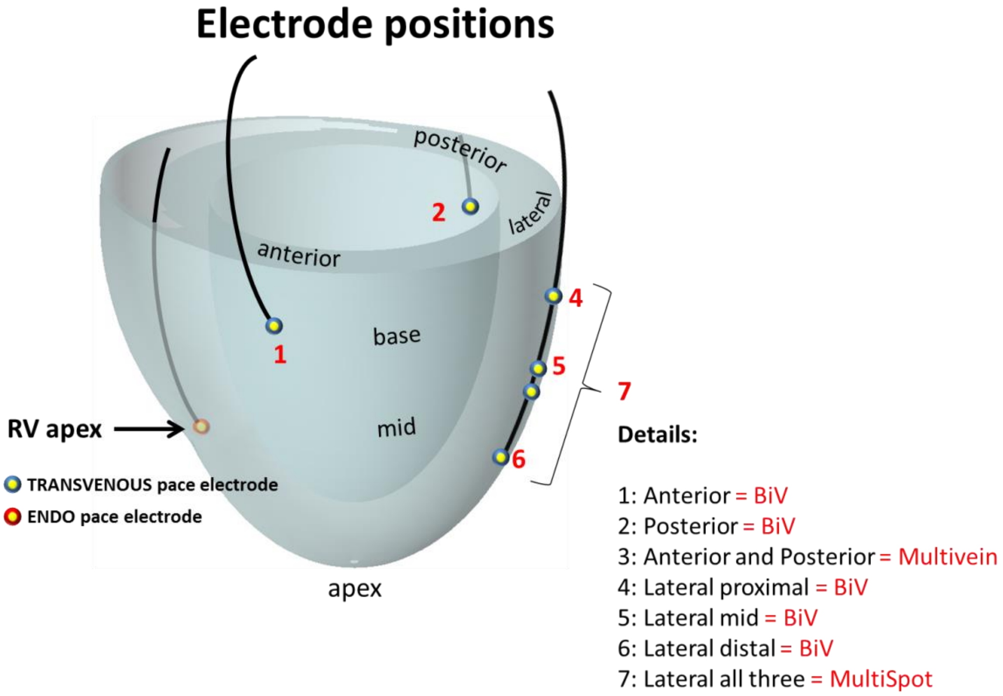

Figure 1: Figure depicting the different ventricular lead positions*. Only two coronary sinus leads will be placed simultaneously. The EP-catheter used for the (postero)lateral coronary vein may be repositioned in the posterior coronary vein or vice versa. Pacing protocol includes synchronized pacing from the RV and LV electrode(s). Up to three "spots" in the LV lateral vein will be paced simultaneously.*

The veins will be accessed using EP-catheters or currently used coronary sinus over the wire (OTW)-pacing leads. Once the three configurations have been evaluated the investigational procedures will end and the EP/CRT-implant-procedure will continue as per standard practice.
Non-invasive sensor data include the measurements of arterial pressure performed by finger plethysmography and the surface ECG. Invasive sensor data will be derived from the electrocardiogram timing, i.e. the electrical delay between the RV and/or different LV sites from the implanted leads/catheters. These measurements will be correlated with positive LV dP/dt max to determine the feasibility of using them to guide LV lead placement. Optional, also invasive blood pressure will be collected.

3 Study Background and justification

CRT has been one of the most important advancements in the past decade for patients with systolic heart failure (HF) and a wide QRS. Several clinical trials have shown improvements in mortality [1, 2], exercise capacity [1], clinical symptoms [2, 3], and quality of life (QOL). However, not all patients improve. Frequently, a positive response rate (many definitions have been used in clinical trials including: LV dP/dt max or LV volumes) of 70% is quoted. An analysis of various definitions of response showed response rates ranging from 32% to 91%. [4].

Multiple reasons for such a substantial non-responder rate have been proposed, such as underlying HF severity and etiology, suboptimal device programming, inadequate viable myocardium, lack of baseline dyssynchrony, and LV lead position [1-3]. With regard to the latter, results from retrospective studies have suggested (1) that LV lead placement at the lateral or (postero)-lateral position displayed a higher response rate to CRT than patients in whom the lead is placed at other sites [5, 6] and (2) that apical LV lead placement also appears to be inferior to a mid-ventricular or basal location [7]. However, in a small study of 24 patients, Gold showed that there were no significant differences in the response to CRT, as measured by acute measurement of positive LV dP/dt max, between basal and apical pacing sites, and there was significant variability between optimal sites among individuals [8].
Currently, research aims to maximize the hemodynamic response within each patient preferentially using a general approach. Several approaches are under development. However, most of the studies mentioned below need to be interpreted with care since they are single-center, and small patient-numbered investigations, only investigating one new experimental approach for increased CRT-response. The proposed multi-centered study (iSPOT) will within each patient simultaneously assess and compare 3 different lead configurations, use multiple repetitions to reduce inherent measurement variability and assess different AV-delay on cardiac dynamics.

Recent efforts to increase the responder rate and response level is to pace from multiple sites [9]. Multisite pacing can be achieved either by the introduction of a second LV or RV lead [10-15] or by additional electrodes on one lead (Quadripolar system) [16-18].
In a study by Leclercq and coworkers, patients presenting with advanced CHF and permanent AF were implanted with one LV lead into a postero-lateral or lateral vein and a second LV lead as far as possible from the first lead [12]. After 3 months of follow-up, patients randomized to dual LV pacing had significantly higher LV ejection fraction and smaller LV end-systolic volumes than those randomized to standard BiV (single LV) pacing in the postero-lateral or lateral vein. However, there was no difference in the primary endpoints of QOL and 6-MHW. More studies with the addition of a second LV lead are underway [11, 13, 14]. In the proposed study, the Multivein configuration will have maximal separation of the pacing spots in the circumferential direction and capture a big area of the left ventricle which could reflect in better CRT response.
The use of one-lead with multiple electrodes has recently been investigated in more detail with regard to improving (hemodynamic) response in CRT patients. Clear benefit was shown in avoidance of phrenic nerve stimulation and the possibility to choose better pacing thresholds [17, 19, 20]. In our study, the Multivein configuration will be avoiding phrenic nerve stimulation because of the nerve's anatomical location. Preliminary results more focusing on the direct measurement of acute hemodynamic response (positive LV dP/dt max) have also shown that within one vein different hemodynamic response is achieved [21]. From earlier studies it was shown that apical LV pacing leads to more hospitalizations and death when compared to basal LV pacing [22]. This may discourage pacing from the more distal electrodes. However, the possibility now to pace from multi-spots within one vein (either simultaneously or sequentially) provides more possibilities and possibly increased patient benefit. However, very recently, it has been shown that the acute hemodynamic response within one vein is not enhanced by using more spots simultaneously

[23]. So, the current evidence for a better hemodynamic response using more spots, in the longitudinal direction, on one lead to pace from might be small.

In addition to the hemodynamic response achieved by each LV pacing configuration procedure complexity and safety has to be considered. While a two LV-lead system (i.e. one in posterior and one in the anterior vein) could provide more circumferential tissue to be activated, low occurrence of phrenic nerve stimulation, less dispersion of repolarization, less dependence on the location of the conduction block and less interventricular asynchrony, the disadvantages are the more complicated implant procedure, increased risk for lead complications and the higher battery usage.
In comparison, the LV Multispot-lead systems may be easier to implant and be associated with less lead complications. However, recently the importance of a two LV lead system was compared to a Multispot configuration [24]. Multispot pacing alone did not improve the positive dP/dt max when compared to standard BiV pacing, but Quadripolar pacing using an additional LV lead did increase positive dP/dt max. The latter pacing method is comparable to the two vein approach in the current study. These results are indicative that coronary vein choice or choices is a critical determinant in CRT-patients.

In this study we will evaluate three different configurations (BiV, Multivein and, Multispot) and hypothesize that the positive LV dP/dt max achieved by the one-lead multiple electrode configuration (Multispot) or two-vein configuration (Multivein) will be similar to that achieved by the current standard BiV lead configuration (non-inferiority).

Despite positive LV dP/dt max being a good predictor of acute and long-term clinical benefit [16, 25], routine clinical use of a LV pressure catheter to guide LV lead placement is unlikely during the implantation of an LV lead. This is due to the additional time and risk of the left heart catheterization. A non-invasive sensor to guide the different LV lead or leads placement would likely find greater acceptance. A number of non-invasive sensors have been used for optimization of CRT device parameters including finger plethysmography, [26-28], impedance cardiography [29, 30] and electrocardiographic imaging [31]. For this study, we investigate if different measures e.g. stroke volume by finger plethsymography, or QRST integral maps by surface mapping [32] can be useful to assess the potential benefit of two-LV leads. Justification for the study is that the results may improve our understanding of CRT lead placement and provide objective data comparing three different lead configurations on a per patient basis. The study will also evaluate the feasibility of non-invasive sensors to assist with optimal lead placement. Additionally, it is anticipated that this study will provide data that can be used to design future studies.

4 System description and intended use

4.1 Pacing devices

In order to deliver pacing on 4 different regions on the heart a system was developed which consists of a “master” pacer and four “slaves” pacers (Figure 2). The master pacer is the 2290 analyzer (in the 2090 Programmer) which will be used to pace the right atrium. The other pacing output (i.e. the ventricular) of the 2290 will be used as an atrial input for the up to four “slaves”. The “slaves”

are used to pace the right ventricle and up to three left ventricular spots. The master pacer can be used to adjust AV-delay for all “slaves”. The “slaves” are hand-held pacers (Medtronic model number: 5388 DDD) and can be programmed independently. In this study the “slaves” will be programmed to specific settings before start of the experimental protocol. During the entire protocol the “slaves” will be either turned “on” or “off”. No additional intra-protocol settings will be changed. Benchmark testing performed has found that upon simultaneous pacing, i.e using all four “slaves” the delay between them was ~1 ms (range 0-2 ms).

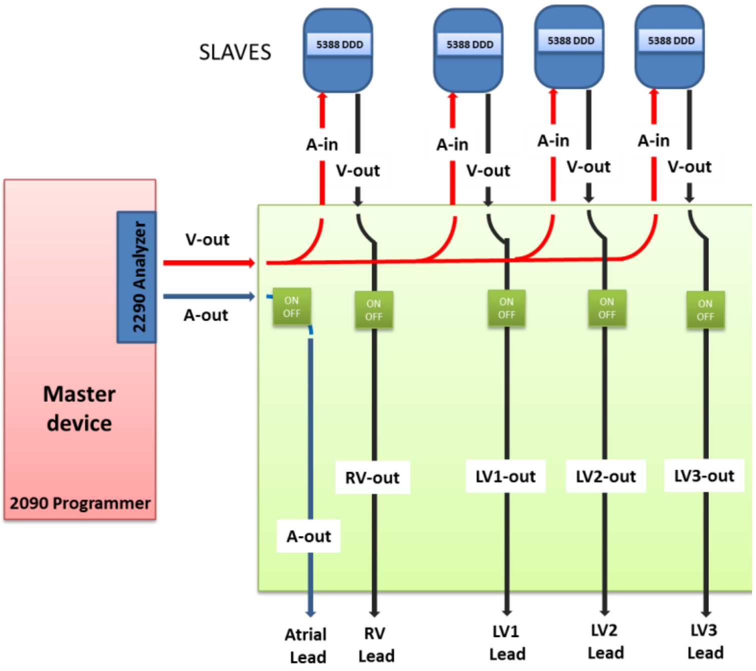

Figure 2: iSPOT equipment set-up. *Green depicts the connection box. For more explanation see text.*

4.2 Leads and electrophysiological catheters

Only commercial market released leads and electrophysiological catheters will be used within their intended use to test the feasibility of multispot pacing for increased acute hemodynamic response to CRT. In this study we will use the Medtronic (TORQR CS or Soloist) intra-cardiac electrode catheters for the delivery of multispot pacing. These catheters are designed for recording intracardiac electrograms and temporary pacing associated with electrophysiological studies (Figure 3). For adequate delivery of these EP-catheters into the coronary veins specially designed Medronic tools can be used (ATTAIN SELECT II and ATTAIN). For the part of the study which requires two separate leads/catheters, standard Medtronic over-the-wire passive leads will be used (e.g. 4196) in addition to the possible replacement from a lateral position to a more anterior or posterior position of the EP-catheters.

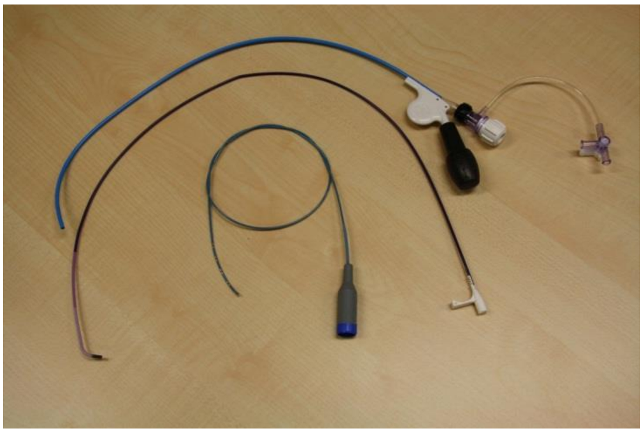

Figure 3: Multispot electrophysiological catheters. *Lower catheter is de TORQR-CS then going up is the ATTAIN for guidance into the specific coronary veins and top is the ATTAIN SELECT II for guidance to the ostium of the coronary sinus.*

4.3 Acquisition system

All data–acquisition will be done using a multi-channel ambulatory system for (electro)-physiological research (porti-system by TMSI). The 32-channel Porti is connected to a PC by means of a bi-directional glass fiber. It will be possible to time-aligned acquire all parameters measured in our study. This includes up to 7 bipolar or unipolar electrograms (EGMs), a 12-lead electrocardiograms (ECG) and up to 3 pressure signals. After a recording is ended the PC reads out the memory card, making the measurement data available for further analysis. During the entire experimental protocol, the following will be measured continuously:

- Unipolar/bipolar EGMs for all the different leads in the heart (if not paced)
- Multi-lead ECG and vectorcardiogram
- Ventricular pressure (using a left ventricular Millar pressure catheter)
- (Optional) Non-invasive blood pressure (using finger plethysmography) and
- invasive blood pressure (through an arterial access line available because of LV pressure measurement)

The devices that will be involved in the conduct of this study are listed in Table 3. Medtronic may incorporate additional Medtronic devices, programmers, software and accessories into this clinical study as they receive appropriate license or regulatory approval or/and are commercially released by Medtronic, providing the scientific soundness of the study is not adversely affected.

**Table 3: Devices and accessories**		
**Component**	**Model Number**	**Investigational or market released**
Medtronic hand-held pacer	5388 DDD	Commercially Released
Leads (endocardial or transvenous leads)	Atrial lead<br>RV lead<br>LV lead	Commercially Released
Left ventricular pressure wires	Millar<br>825-0101	Commercially Released
Blood pressure	Recommended: Codan<br>PVB transducer 17.8059 and contact board 75.1068.00	Commercially Released
Medtronic-EP catheters	Soloist<br>TORQR-CS<br>041865CS<br>041590CS<br>041565CS	Commercially Released
Medtronic- Guiding catheters	ATTAIN 6250S 6250C<br>6227DEF<br>ATTAIN SELECT II<br>6238TEL<br>6248DEL	Commercially Released
CareLink® Programmer	2090	Commercially Released
Analyzer	2290	Commercially Released

Component	Model Number	Investigational or market released
Surgical connection cables	5114<br>5832(S)<br>5833<br>5487	Commercially Released
Adaptor	5103	Commercially Released
Reference lead	Recommended:<br>F7830S<br>F7832S	Commercially Released
Catheter connecting cable, 10 conductor	05518SP	Commercially Released
Connection Box and connectors	09083	Investigational

The connection box is investigational. All other products used in the present study (leads and pacing devices) are commercially released (and CE-marked) and used within their intended purpose.

More detail on the basic components of the system:

The Medtronic TORQR or Soloist EP catheters are intended for use in diagnostic EP procedures. The catheter is designed for recording intracardiac electrograms and temporary pacing associated with electrophysiology studies.

The Medtronic Model 5388 DDD hand-held pacemaker is a dual chamber temporary pacemaker. A dual pacemaker has the ability to sense and pace in both chambers of the heart.

The Medtronic Analyzer is intended for use by a clinician to analyze the pacing and sensing performance of the cardiac lead system during the implant of a cardiac arrhythmia management device, or during invasive troubleshooting of a cardiac lead system.

Reference lead: Extension cable for threshold analysis for permanent or temporary cardiac pacing

05518SP: Connection cable is used during electrophysiological studies to connect electrode catheter to ECG recording/stimulation equipment.

825-0101: Pressure wire: The Mikro-Cath is a single-use cardiovascular catheter intended to be used for medical research and diagnostic purposes. The catheter is used to measure hemodynamic cardiac pressures in the human body to allow physicians to better understand cardiac performance.

DPT9003: Invasive blood pressure monitoring. The sample line of the sheet used to guide the left ventricular pressure wire is connected to the xtrans pressure transducer.

The Model 09083 Connection Box:
The Medtronic Model 09083 Connection Box connects pacing electrodes or temporary stimulation electrodes to external pacemakers during the pre-implant EP testing procedure. The connection box allows easy, flexible and safe connection of pacing electrodes to external stimulators, while simultaneously offering the possibility to feed electrocardiogram signals obtained from the pacing electrodes into an electrophysiological monitoring and recording system. The connection box contains six channels. Each channel can be individually switched on or off, connecting the stimulator to the connected pacing electrode, as needed. Each of the six channels can be switched to unipolar of bipolar cardiac stimulation independently.

The connection box has been designed to facilitate connection during the EP pre-Implant testing procedure, when multiple electrodes are used. The connection box improves the management of the intervention and helps thereby to minimize the prolongation of the procedure and improve safety. Additionally the connection box helps to minimize the likeli hood of arrhythmias as a result of setups.
The connection box is designed such that the set up intuitive. Connections to the monitoring system are located on one side, all connections to external pacemakers are located at another side of the box, and all connections towards the stimulation electrodes are located at a third site of the device. All switches are placed on the top of the device. Switches are consistently physically linked to the switched stimulation channel, connecting an external stimulator and a stimulation electrode.

All the recommended devices connecting to the connection box have CE-mark with clear defined functionality and electrical standards. Inserting the connection box into the electrical pathway does not significantly change the electrical characteristics (impedance, capacitance) of the pacing system.

The connection box will be accompanied with cabling to connect to recommended Medtronic equipment. The box nor its pacing connection cables are in direct contact with the patient, or will be in the sterile patient field, and do not need to be sterilized.

The intended use of the connection box is to facilitate, the CRT pre-implant physiological testing procedure, involving multiple cardiac stimulation sites in the right and/or left ventricle. The box facilitates the interconnection between pacing devices and selected pacing locations in the patient’s right and/or left ventricle. It increases the safety of the patient while undergoing the procedure and will only indirectly (through a specific clinical protocol) aid in the diagnosis. The device is reusable, non-invasive, non-sterile box with no direct contact to the patient. The box will not be used in a magnetic resonance imaging (MRI) environment.

For all other products the manual can be used for review. Labeling is available in local languages.

5 Regulatory compliance

The iSPOT clinical study is an exploratory, prospective, interventional non-randomized multi-center research study. The study will be conducted according to the Data Protection Directive, the Active Implantable Medical Device Directive (AIMDD), Declaration of Helsinki, good clinical practice (GCP), this Clinical Investigation Plan (CIP), national and local laws, regulations, standards, and requirements of the countries/geographies where the study is being conducted. Furthermore, the study is conducted in line with ISO 14155:2011, except for the site initiation visit, IB acknowledgement, financial disclosure collection, monitoring, and equipment maintenance and calibration. Prior approval of the CIP and any subsequent amendments is required from the following groups prior to any study procedures at a study center: Medtronic, geography-specific regulatory authorities (if regulatory approval is required) and an independent medical ethics committee or institutional review board.

The principles of the Declaration of Helsinki are implemented in this study by means of the Patient Informed Consent process, MEC approval, study training, pre-clinical testing, clinical trial registration, publication policy, etc.

6 Methodology

6.1 Study Design

This clinical trial is a multi-center, exploratory, prospective, interventional, non-randomized study designed to assess augmentation of contractility as measured by positive LV dP/dt max across LV pacing site(s) in patients indicated for CRT. Thereto, the force of contraction during multivein or multispot pacing is compared to standard BiV pacing. Standard BiV pacing is defined as pacing from a (postero-)laterally placed lead with the electrode between the base and mid position of the left ventricle with optimized AV delay and VV-delay is zero. In addition, the feasibility of (non)invasive sensors to identify this site(s) of maximal positive dP/dt max values is assessed. Rationale for study design is to aim for maximizing the acute hemodynamic response on a per patient basis.

6.2 Study Objectives

6.2.1 Primary Objective

The primary objective of this study is to compare the hemodynamic response of a multispot LV pacing configuration to a single spot LV pacing configuration (BiV) in patients undergoing a research study, an EP exploratory procedure or CRT-implant using the contractility parameter positive LV dP/dt max.

6.2.2 Secondary Objectives

The secondary objectives of this study are to:

- Compare the positive LV dP/dt max from the multi-vein LV pacing configuration to the standard single spot LV pacing.
- Compare the positive LV dP/dt max from the multi-vein LV pacing configuration to the multispot LV pacing.

- Correlate the (non)invasive measures (blood pressure, electrocardiographic mapping and RV/LV EGM timings) obtained during the three pacing configuration's to the positive LV dP/dt max measures obtained.
- Evaluate the non-invasive measures ability to identify the pacing configuration with the highest positive LV dP/dt max.
- Evaluate the within patient variability in positive LV dP/dt max measures.

6.3 Subject Selection

The study is expected to be conducted in approximately 8 centers in Europe and Middle East and Africa (MEA). The study will enroll up to 40 subjects which are required to complete study procedures. It is anticipated this study will require approximately 12 to 18 months for subject enrollment. Patients' participation in this study is expected to last approximately between 1 day and 3 months, pending on the time between enrollment and the EP study and the duration of hospital stay after the research study, an EP related procedure, or the CRT-implant. Subjects must meet all of the inclusion criteria and none of the exclusion criteria to be included in the study.

6.3.1 Inclusion Criteria

- Subject is indicated for CRT or CRT-D device according to current applicable ESC/AHA guidelines
- Subject has a left bundle branch block (LBBB) conduction pattern
- Subject is in stable sinus rhythm at the time of the EP visit.
- Subject receives optimal heart failure oral medical therapy (ACE inhibitor and/or ARB and Beta Blockers).
- Subject (or the legal guardian) is willing to sign informed consent form
- Subject is 18 years or older or as specified minimal age per local law/regulation

6.3.2 Exclusion Criteria

- Subject has permanent atrial fibrillation/ flutter or tachycardia
- Subject experienced recent myocardial infarction (MI), within 40 days prior to enrollment.
- Subject underwent coronary artery bypass graft (CABG) or valve surgery, within 90 days prior to enrollment
- Subject is post heart transplantation, or is actively listed on the transplantation list
- Subject is implanted with a left ventricular assist device (LVAD)
- Subject has severe renal disease (up to physicians discretion)
- Subject is on continuous or uninterrupted infusion (inotropic) therapy for heart failure (≥ 2 stable infusions per week)
- Subject has severe aortic stenosis (with a valve area of <1.0 cm^2 or significant valve disease expected to be operated within study period)
- Subject has complex and uncorrected congenital heart disease
- Subject has a mechanical heart valve

- Pregnant or breastfeeding women, or women of child bearing potential and who are not on a reliable form of birth control
- Subject is enrolled in one or more concurrent studies that would confound the results of this study
-

6.4 Minimization of bias

Potential sources of bias in this study may result from selection of subjects, treatment of subjects, and evaluation of study data. Methods incorporated in the study design to minimize potential bias include but are not limited to:

- Subjects will be their own control, and study measurements have been designed to minimize the impact of within-subject temporal variation.
- Subjects will be screened to confirm eligibility for enrollment with defined inclusion/exclusion criteria prior to enrollment (See Section 6.3 Subject Selection)
- Subject demographics will be collected at the enrollment visit on possible differences that may affect primary endpoints.
- Data collection requirements and study procedures will be standardized across all study centers, including identical and calibrated equipment.
- All implanters in the study will be experienced in the placement of LV leads, performing EP procedures, performing LV pressure measurements.
- All study center personnel and Medtronic personnel will be trained on their respective aspects of the study using standardized training materials. All study clinicians will be trained on and required to follow the Clinical Investigational Plan.

In summary, potential sources of bias that may be encountered in this clinical investigation have been considered and minimized by careful study design.

6.5 Data Monitoring Committee

A Data Monitoring Committee will not be utilized for this study considering:

- As a result of risk analysis and mitigation efforts as outlined in Section 11, any residual risk associated with this study is considered low and acceptable.
- All products used in the study (leads and pacing devices) are commercially released (and CE-marked) and used within their intended purpose, except for the Connection Box which is investigational.

An independent Adverse Event Adjudication Committee (AEAC) will be utilized to review and classify the relatedness and severity of all events and deaths.

7 Study Visits and Procedures

7.1 Center/Investigator selection criteria

All centers that participate in the iSPOT study have to comply with the following minimum requirements:

- Investigator has education / experience in the field of the research (EP procedures and CRT- pacing)
- The center has adequate resources, facilities and equipment at the center to meet the expected enrollment rate.
- The investigator/center has a potential subject population and will be able to enroll approximately 8 subjects in the study
- Investigator has experience in conducting clinical research trials

7.2 Center activation

All local and regional regulatory requirements will be fulfilled prior to center activation and enrollment of subjects into the study. Each study site must have written documentation of site and investigator readiness, including (but not limited to):

- Ethics Board approval for the current version of the CIP and Patient Informed Consent (PIC)
- Evidence that the investigator did not participate in the MEC approval
- Competent Authority (CA) approval or notification (if required)
- Current (Co-)Investigator Curriculum Vitae (CV) on file with sponsor (signed and dated)
- Signed/dated Clinical Trial Agreement (CTA) on file with sponsor
- Signed/dated Financial Disclosure form (if needed per country requirements)
- Signed/dated documentation of training of required study personnel

Prior to investigational site activation or subsequent involvement in study activities, Medtronic will provide study training relevant and pertinent to the involvement of personnel conducting study activities and investigator responsibilities. Training will be given on the CIP, the Informed Consent (IC) process, the use of data collection tools, and the database. Study materials will be provided to the site upon investigational site activation.

Medtronic will provide each study center with written documentation of study center/investigator readiness, this letter must be received prior to subject enrollment.

Investigator and/or study coordinator meeting(s) or conference call(s) may be held to discuss the CIP, provide training, discuss study results, etc. Continued training may occur through interim meetings or telephone conference calls to discuss relevant study issues.

7.2.1 Equipment Requirements

The following study equipment must be available at each center to support study activities:

- Computer with high speed internet access and Microsoft Internet Explorer available for data entry
- Market released Medtronic programmer (Model 2090)
- Pacing System Analyzer (Model 2290)
- MRI scanner

It is preferable that the following study equipment is available at a center to support study activities:

- Non-invasive beat-to-beat blood pressure monitor (such as Finapres, Finapres Medical Systems, Amsterdam, the Netherlands; Nexfin, Bmeye, Amsterdam, The Netherlands, or similar).

All study equipment must be properly maintained and calibrated if required. Programmer calibration and maintenance will be performed at the Medtronic office in Heerlen, The Netherlands. Only calibrated programmers may be used. Centers are responsible for maintaining and calibrating other equipment not provided by Medtronic and used in the course of this study in accordance with established center practice.

7.3 Patient Informed Consent Process

The Informed Consent (IC) process is defined as the process by which an individual is provided information and is asked to voluntarily participate in a clinical investigation (ISO 14155:2011). The Consent Form (CF) is defined as legally effective, documented confirmation of a subject's (or their legally authorized representative) voluntary agreement to participate in a particular clinical investigation after information has been given to the subject on all aspects of the clinical investigation that are relevant to the subject's decision to participate.

At a minimum, each investigational center's Ethics Board will be required to approve the CIP, Investigator's Brochure (IB) and the CF, and any other written study information to be provided to the subjects.

Any changes to a Medtronic and Ethics Board approved version of the CF must be approved by Medtronic and the Ethics Board reviewing the application before being used to consent a prospective study subject. The document(s) should be controlled (i.e. versioned and/or dated) to ensure it is clear which version(s) were approved by the Ethics Board and Medtronic.

Prior to initiation of any study-specific procedures, subjects (or their legally authorized representative) must sign and date the Ethics Board and Medtronic approved CF. The CF must be given to the subject (or their legally authorized representative) in a language he/she is able to read and understand. The principal investigator or authorized designee also must personally sign and date the CF. The process of IC will not be conducted using coercion, or undue influence by the investigator or other center personnel.

The investigator must notify the subject of any significant new findings and answer all questions to the satisfaction of the subject.

The process of obtaining IC shall:

- ensure that the principal investigator or his/her authorized designee conducts the IC process,
- include all aspects of the clinical investigation that are relevant to the subject's decision to participate throughout the clinical investigation,
- avoid any coercion or undue improper influence on, or inducement of, the subject to participate,
- not waive or appear to waive the subject's legal rights,
- use native, non-technical language that is understandable to the subject,
- provide ample time for the subject to read and understand the CF and to consider participating in the clinical investigation,
- include personally dated signatures of the subject or legally authorized representative,
- provide the subject with a copy of the signed and dated CF and any other written information,
- show how IC will be obtained and recorded in circumstances where the subject is unable to provide it him- or herself
- ensure important new information is provided to new and existing subjects throughout the clinical investigation.

The process of IC will not be conducted using coercion, or undue influence by the investigator or other center personnel. The CF must be obtained through a supervised oral process and an independent witness must be present throughout the entire process in the event the patient is not able to read and/or write. The information must be read aloud and explained to the subject. Whenever possible, the subject must also sign and date the IC to attest that the information was accurately explained and that informed consent was freely given.

In the event the subject cannot read and/or write, witnessed (impartial third party) IC will be allowed, provided detailed documentation of the process is recorded in the subject’s case history and the witness signs and dates the CF. The original or a copy of the signed CF must be filed in the hospital/clinical chart or with the subject’s study documents.

The investigator must document the subject’s participation in the medical record. If the IC is obtained the same day the subject begins participating in study-related procedures, it is recommended that it is documented that consent was obtained prior to participation in any study-related procedures.

The CF must be available for monitoring and auditing. Any Medtronic Field personnel who support the study visit must be allowed to review the subject’s signed and dated CF and verify its completeness prior to proceeding with the study visit. In the event the Medtronic Field personnel identify a CF as being incomplete, the study visit will not be allowed to occur until the consent of the subject can be adequately and appropriately obtained.

7.4 Study visits

This section explains which visits are required, when they should occur and what study procedures should be performed during each visit.

7.4.1 Baseline visit

The baseline visit can be a stand alone visit or occur on the same day as the EP study. A subject is enrolled in the study when he/she signs the Informed Consent Form.

At this point, the patient will be assigned a subject number and is considered a subject in the investigation. The following procedures/data collection will be conducted during the baseline visit:

- Verification of all inclusion and all exclusion criteria
- Enrollment
- Subject demographics (e.g., age, gender, height, weight, race)
- Medical history (cardiovascular or related)
- Cardiovascular medications
- NYHA classification
- Blood pressure
- Left ventricular ejection fraction (within last 3 months, otherwise a new measurement should be performed)
- Multi-lead ECG (within last 3 months, otherwise a new ECG should be performed)
- Pregnancy test for women with child-bearing potential
- Echocardiographic evaluation (if available within last 3 months, otherwise not collected)
- Venous blood sample to determine heart failure severity (NTproBNP, creatinine and hemoglobin)
- Magnetic Resonance Imaging (MRI) as explained in paragraph below

7.4.1.1 Magnetic Resonance Imaging (MRI)

Patients will undergo a baseline MRI before scheduled CRT-related surgery (i.e. is dedicated research study or EP procedure or actual CRT implant) to identify the location and transmurality of any myocardial scar using late gadolinium enhancement-magnetic resonance imaging according to standard hospital practice. In addition, left ventricular end systolic volume (LVESV), left ventricular end diastolic volume (LVEDV) and left ventricular ejection fraction (LVEF) will be determined. MRI can be performed as standard of care or solely for the study.

7.4.2 Electrophysiological (EP) study visit

The EP study visit can be a stand alone visit or can occur on the same day as the baseline visit. The EP study visit should take place within 3 months after the MRI has been obtained. Patients will undergo standard implant of a biventricular pacing system (Medtronic CRT or CRT-D) or will undergo an EP study before possible CRT-implant. If available in the center, a Finger plethysmography system (eg. model Nexfin, BMEYE B.V.) will be connected to one of the subject’s fingers to measure the blood pressure non-invasively. In addition, a multi-lead surface ECG is collected during the whole procedure.

Pacing electrodes (cardiac leads or EP catheters) will be placed in the right atrium (RA) and right ventricle (RV). The coronary sinus will be cannulated, a venogram will be obtained and target vessels for LV stimulation will be identified. An EP-catheter (“Multi-electrode lead”) or LV lead will be used for LV pacing. Also a CE-certified LV-quadripolar lead from Medtronic, when available, can be used in the study as an alternative for the EP-catheter.The temporary LV pacing electrodes (EP catheter or LV lead) will be inserted into the coronary sinus (CS) and then sequentially into each suitable target vein.

Targeted myocardial regions for LV lead/catheter placement are: lateral or posterolateral (Standard vein), posterior (Alternative vein I) and anterior (Alternative vein II). Pacing will be performed at up to three spots simultaneously within the Standard vein - MultiSpot), covering the basal to apical locations when feasible (see figure 4). The leads placed in the alternative veins I and II will be stimulated separately or simultaneously (multi-vein; uni-spot) covering the heart circumferentially. A lateral LAO, RAO and/or AP fluoro-screen shot will be taken to document the different (temporary) lead positions.

A Millar pressure catheter or similar will be introduced via the femoral artery retrograde across the aortic valve into the LV for hemodynamic pressure monitoring. Heparin will be administered to ensure that the activated clotting time is monitored per standard hospital practice. The different pacing settings include simultaneous RV- and LV pacing and are in bipolar or unipolar mode.

The MRI-images will ascertain avoidance of pacing ischemic scar tissue. In addition, paced left ventricular QRS-width may also be used for optimal coronary sinus lead placement [33] .

A

B

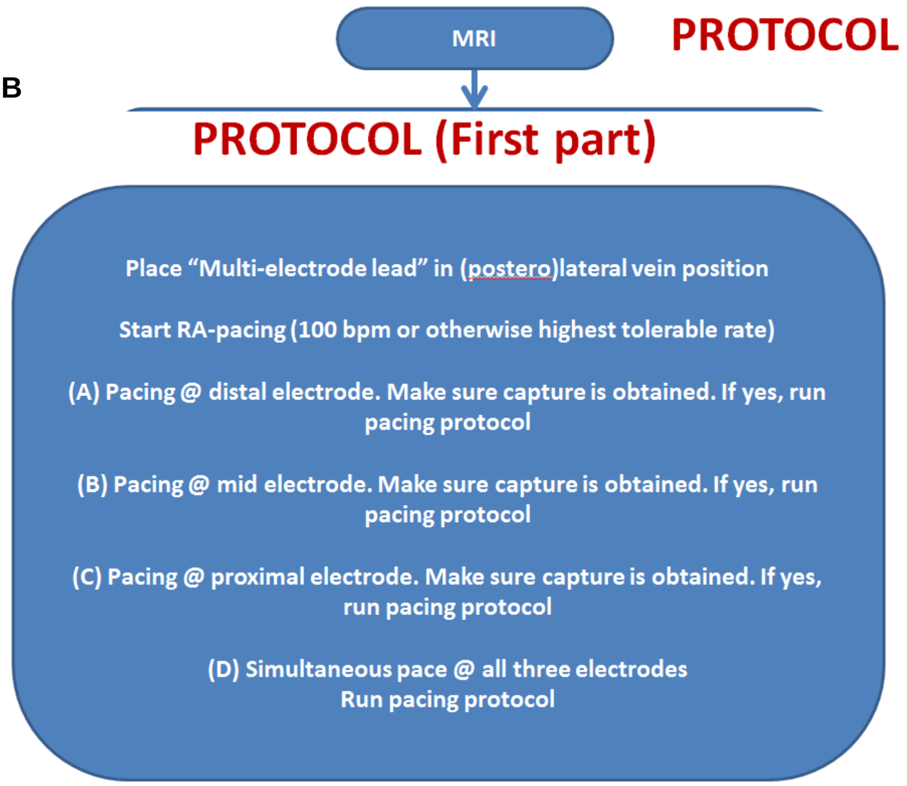
MRI
PROTOCOL
PROTOCOL (First part)
Place "Multi-electrode lead" in (postero)lateral vein position
Start RA-pacing (100 bpm or otherwise highest tolerable rate)
(A) Pacing @ distal electrode. Make sure capture is obtained. If yes, run pacing protocol
(B) Pacing @ mid electrode. Make sure capture is obtained. If yes, run pacing protocol
(C) Pacing @ proximal electrode. Make sure capture is obtained. If yes, run pacing protocol
(D) Simultaneous pace @ all three electrodes
Run pacing protocol

C

PROTOCOL (Second part)

RePlace "Multi-electrode lead" in anterior vein position
Add a second lead in the posterior vein

Start RA-pacing (100 bpm or otherwise highest tolerable rate)

(A) Pacing @ distal electrode. Make sure capture is obtained. If yes, run pacing protocol

(B) Pacing @ mid electrode. Make sure capture is obtained. If yes, run pacing protocol

(C) Pacing @ proximal electrode. Make sure capture is obtained. If yes, run pacing protocol

(D) Simultaneous pace @ all three electrodes
Run pacing protocol

D

PROTOCOL (Alternative Second part)

Leave "Multi-electrode lead" (postero)latereral vein position
Add a second lead in the posterior or anterior vein

Start RA-pacing (100 bpm or otherwise highest tolerable rate)

(A) Pacing @ distal electrode. Make sure capture is obtained. If yes, run pacing protocol

(B) Pacing @ mid electrode. Make sure capture is obtained. If yes, run pacing protocol

(C) Pacing @ proximal electrode. Make sure capture is obtained. If yes, run pacing protocol

(D) Simultaneous pace @ all three electrodes
Run pacing protocol

E Pacing protocol

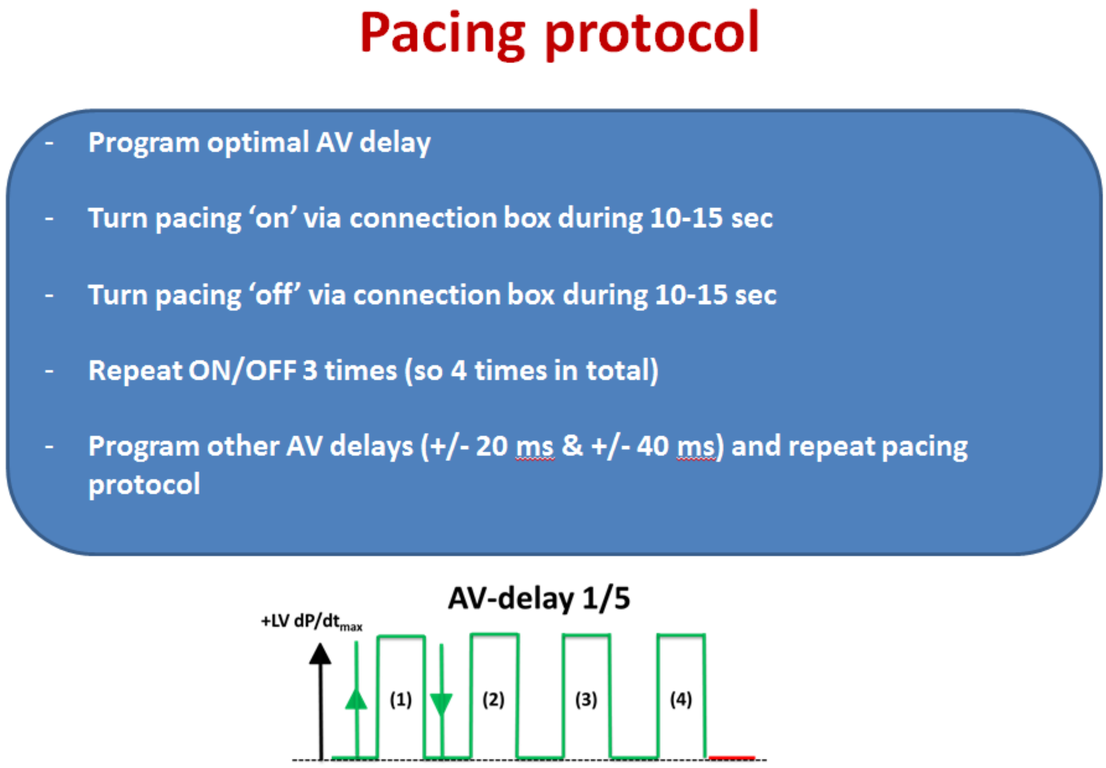

Figure 4: Description of the pacing protocol. *In A the start of the protocol is depicted which involves imaging of the venous anatomy of the patient. The protocol can be separated into two parts, depending on the patient's anatomy. Part B is similar for every patient while part C or D is patient-dependent. In general, one part consists of BiV or Multivein using one or both of two opposite coronary veins (multivein approach). The other part consists of using up to three LV electrodes (BiV or QuadV) to pace from the (postero)lateral vein (multispot approach). Part E is displaying the pacing procedure for 1 out of total 5 AV delays during which the positive dP/dt max will be measured. Each setting, as indicated by a horizontal line in the figure, will last 10-15 sec. The repetition of steps is used to increase sensitivity of the positive dP/dt max measurements. Five AV delays will be assessed: optimal AV, optimal AV delay +/- 20 and +/-40 ms.*

At each pacing site, the evaluation of the effect of CRT will be performed approximately 4 times (repetition) in order to increase signal to noise ratio. Each setting lasts about 20 beats (10-15 sec) interspersed with baseline (AAI; also 20 beats, 10-15 sec) pacing. The heart rate will be elevated by atrial pacing to a base rate to 100 bpm or maximally tolerable rate (less than 10% drop in dP/dt max from baseline no pace), to ensure a constant heart rate. The RV-LV (= VV-timing) intervals will be zero. Optimal AV-delay will be calculated using formulas (PAV_{ECG}=min {Ap-Pend + 30 ms, Ap-RVs – 50 ms}) derived

from a correlation study between electrocardiographic measures such as the intrinsic AV interval and P-wave duration. The method aims at absence of truncation of the E/A waves. Up to five different AV-delays will be evaluated in this study (i.e. the optimal AV-delay and optimal AV-delay +/- 20 and +/- 40 ms). Hemodynamic measurements of positive LV dP/dt max, LV End diastolic pressure, arterial pressures, and stroke volume derived from arterial pressure will be measured during baseline and CRT. Additionally, at each pacing setting, the electrical delay from RV-pace to the different LV activations will be measured.

The signals from the LV pressure catheter, finger plethysmography device, internal electrograms, and surface ECG will be collected in real time and stored for later analysis as explained earlier (section 4.3). The CRT implant or EP-procedure will be completed as per standard practice.

Protocol procedures are anticipated to last about 60 to 120 minutes. Pacing will be achieved by using a Medtronic 2290 analyzer and temporary pacemaker (5388) stimulators or equipment present in the OR of the participating physicians.

Sponsor representatives (e.g. Field Clinical Engineer, Field CRS, Technical Consultant) may be delegated to perform certain study related activities such as technical support during procedures and data collection. Documentation of study training and qualification of sponsor representatives will be maintained by Medtronic.

7.5 Subject Exit and Withdrawal

Study Exit is defined as the moment when a subject officially stops participating in the study. The date and reason for subject exit must be reported to Medtronic at the earliest opportunity.

Reasons subject may be exited form the study include, but are not limited to:

- Study completion
- Subject did not provide consent
- Subject did not meet inclusion/exclusion criteria
- Subject chooses to withdraw (e.g., consent withdrawals, relocation to another geographic location)
- Investigator deems withdrawal necessary (e.g., medically justified, inclusion/exclusion criteria not met, failure of subject to maintain adequate study compliance)
- Subject death

Subjects will be exited from the study just before the moment of hospital discharge. This will be documented on the Study Exit CRF. There will be no further follow-up required for subjects that are exited form the study since no investigational products will be implanted or used in the study.

7.5.1 Subject Initiated Withdrawal

A subject will be exited from the study in the event that he or she is unable to participate, expresses a desire to withdraw, or is unwilling to continue participation in the study. The reason for withdrawal needs to be documented in the patient chart. The subject should be informed that their future care or treatment will not be affected in any way as a result of choosing to not participate in this study. Furthermore, alternative treatments and medical consequences of exiting the study should be discussed with the subject. Any significant new findings related to the study that may develop, which may relate to the subject's willingness to continue participation, should be communicated to the subject. Unless the patient explicitly revokes informed consent, available data will be reported.

7.5.2 Investigator Initiated Subject Withdrawal

A subject may be exited from the study if an investigator feels it is necessary to withdraw the subject from the study due to a medical condition or other reason. In such cases, the subject will be notified and provided an explanation regarding the reasons for the study exit. Unless the patient explicitly revokes informed consent, available data will be reported.

7.6 Overview of Data Collection Requirements

Clinical data will be collected at enrollment and during the study procedure. Clinical data to be collected may include but is not limited to: demographics, medical history, cardiovascular medications, multi-lead ECG, pregnancy test, digitally stored echo images, digitally stored MRI images, chest X-ray, non-invasive blood pressure monitor data, invasive blood pressure data, LV pressure data, and unipolar/bipolar electrograms. Table 4 summarizes the data requirements.

Table 4: Data Collection Requirements

	Enrollment	Study day
Inclusion/Exclusion Criteria	✓	
Subject Informed Consent	✓	
Demographics, medical history, CV medication, pregnancy test (if applicable)	✓	
MRI (structural and hemodynamic)	✓	
NYHA class	✓	
LVEF	✓	
Surface ECG	✓	✓
Blood chemistry	✓	
Venogram		✓
Non-invasive blood pressure	✓	✓
Invasive blood pressure		✓
LV pressure		✓
LV lead implant location (Chest x-ray)		✓
Unipolar and Bipolar electrograms		✓
Adverse events		✓
Deviation Collection (as indicated, Section	✓	✓
Exit		✓

8 Statistical Method and Data Analysis

8.1 Sample Size Justification

This study is powered for non-inferiority for the primary end point of difference in percentage change dP/dt max from baseline between Biventricular (BiV) pacing and Quadri-ventricular (Multispot) pacing. This is calculated as [percentage change in dP/dt max during Multispot] - [percentage change in dP/dt max during BiV]. Non-inferiority will be declared if the lower limit of a 1-sided 97.5% confidence interval of difference does not fall below -4%. Assuming the expected difference in percentage change dP/dt max between BiV pacing and multispot pacing is 0% and a standard deviation in differences of 8%, 34 patients would have 80% power to demonstrate non-inferiority for dP/dt max. The total sample size is increased to 40 patients to accommodate for non-compliance with protocol. Superiority testing will be performed if non-inferiority is met.

H_0: $\Delta_{multispot} - \Delta_{BiV} \leq -4\%$
H_1: $\Delta_{multispot} - \Delta_{BiV} > -4\%$

Previous literature suggests the average mean difference in percentage change between BiV and no pacing is 18%, in this study we consider Multispot non-inferior to BiV if the difference in their means is less than a quarter of the difference between BiV and no pacing [34]. No literature is available on the standard deviation of differences, which is the variability of the difference between pacing configurations within each subject. However we estimate that it would be less than half the variability seen when comparing differences across different patients.

8.2 Study Endpoint

During the study all hemodynamic and electrophysiological data is continuously collected. The primary endpoint for this study is the positive dP/dt max value during BiV pacing and Multispot pacing. These data will be calculated offline using pre-determined filter-settings and algorithms. The values will be obtained on a beat-to-beat basis. To reduce variance, in particular the positive dP/dtmax values, each pacing setting is repeated 4 times. A similar approach will be taken for the (non)-invasive secondary endpoints.

8.3 Analysis Methods

Descriptive statistics will be used to summarize the patient demographic and clinical characteristics at baseline. Data for qualitative variables will be presented as incidence rates (total number of patients, number of events, and percent). Data for continuous variables will be summarized using measures of central tendency and dispersion.

To calculate the mean LV dP/dt max difference and 95% confidence interval between pacing configurations a restricted maximum likelihood (REML)-based repeated measures approach (MMRM) will be used. Analyses will include the fixed, categorical effects of center (if sufficient number of patients per center available), pacing configuration, AV delay and if possible heartbeat. If the model is unable to support the beat-to-beat dP/dt values (i.e too much variability in the number of beats available per AV delay) then they will be averaged. The within-patient errors will be modelled using an unstructured (co) variance structure. If this analysis fails to converge, auto regressive, toeplitz, compound symmetric, and simple structures, with and without heterogeneous variances by pacing configuration will be tested. The (co)variance structure converging to the best fit, as determined by Akaike's information criterion, will be considered the primary analysis. Significance tests will be based on least-squares means and Type III sum-of-squares. Analyses will be implemented using SAS PROC MIXED.

Correlation coefficients will be calculated for each of the (non)-invasive measures with LV dP/dt, to account for the repeated measurements a linear mixed effects model will be used. Additional, a categorical variable of the best pacing configuration per patient will be calculated (the configuration that achieves the largest dP/dt value) and the agreement with each (non)-invasive

evaluation of best pacing configuration (also derived into a categorical variable) will be evaluated and tested with an unweighted Kappa statistic.

P-values will be evaluated based on a two-sided significance level of 0.05, and interaction effects will be evaluated at a significance level of 0.10. Except for the primary objective which is evaluated using a one-sided significance level of 0.025. No adjustments will be made for multiple testing. No imputation of missing data is planned.

Any change to the data analysis methods described in the Clinical Investigational Plan will require an amendment only if it changes an objective of the Clinical Investigational Plan. Any other change to the data analysis methods described in the Clinical Investigational Plan, and the justification for making the change, will be described in the clinical study report. Additional exploratory analyses of the data will be conducted as deemed appropriate.

8.4 Analysis Populations

All patients who signed the informed consent document will be defined as the All Enrolled Population. The Study Population will be defined as all patients who provide informed consent and complete the study procedures or in who an attempt to start the study procedures is made. The Analysis Population is defined as patients who are enrolled and complete at least two of the three pacing configurations. Patients who are enrolled but are found to have an inclusion or exclusion criteria violation or a significant Clinical Investigational Plan deviation such that the clinical interpretability of the results obtained from the patient is impacted, will not be included in the primary analysis, but will be reported in the patient disposition table. Safety will be reported on the All Enrolled Population and the primary objective will be reported on the Analysis Population.

8.5 Interim Analysis

One interim analysis is planned for this study after approximately 20 patients (one-half) have been enrolled. The primary purpose of this interim analysis is to review safety of study participants and the continuing validity and scientific merit of the study. The primary objective will not be evaluated (i.e the mean difference in pacing configurations will not be calculated) to minimize the operational and statistical bias that may result from performing an interim analysis. However the standard deviation between pacing configurations within patients will be calculated and compared to that used in the sample size assumptions. The study might be re-evaluated in case of deviations from assumed sample size calculations or an increase in the rate of expected adverse events. Study sites will receive information about interim results only if they need to know for the safety of their patients.

9 Data and Quality Management

Study visit data, adverse event data, device deficiency data and study deviations will be collected on electronic case report forms (eCRF) using an electronic data management system for clinical studies. The eCRF data will be reviewed using programmed and manual data checks. Data queries will be

made available to study centers for resolution. The data collected during the EP study visit coming from the heart, chest or finger (i.e. surface ECG, (non-)invasive blood pressure, LV pressure, unipolar and bipolar electrograms) will be collected simultaneously on the universal data acquisition system (i.e. PORTI-system is a multi-channel ambulatory and stationary system for physiological research). The raw data from the PORTI-system will not be processed during the experiment, and no data will be used to guide patient care. The data from the PORTI-system, MRI images, echo images (if available) and venogram will be brought to the Medtronic office by attending Medtronic personnel. A copy of the data will remain at the site. Offline analyses include standard calculations of first time derivative of left ventricular pressure (filter settings), and timing between the different electrodes on the heart or on the chest. All data will be stored in a secure, password-protected database which will be backed up nightly. Study management reports may be generated to monitor data quality and study progress. At the end of the study, the data will be frozen and retained indefinitely by Medtronic.

All data shall be secured against unauthorized access. The privacy of each subject and confidentiality of his/her information shall be preserved in reports and when publishing any data.

Procedures in the CIP require source documentation. Source data is all information in original records, certified copies of original records of clinical findings, observations, or other activities in a clinical study. A source document is a printed, optical or electronic document containing source data. In case a printout is made from an electronic document containing source data, ensure this is a certified copy. A certified copy is signed and dated by study site personnel with a statement that it is a true reproduction of the original source document. In some cases items on the CRFs may be considered source as long as there is evidence of the visit in the subject's record. The eCRF can be source for the following data collection points: the subject number, inclusion criteria "Subject (or the legal guardian) is willing to sign informed consent form", exclusion criteria "Pregnant of breast feeding women, or women of child bearing potential and who are not on a reliable form of birth control" and "Subject is enrolled in one or more concurrent studies that would confound the results of this study", the date the study center became aware of the AE/Death, check boxes on AE CRF for "Prolongation of existing hospitalization" and "Device interrogation", "Device reprograming" on AE CRF, and "Location of subject at time of death" on death CRF. Even when the CRF may be considered as source, an alternate method of source documentation is always strongly encouraged.

More details regarding data management will be described in the Data Management Report.

Appendix A: Study Overview

**Title**	**Left Ventricular MultiSpot Pacing for CRT (iSpot)**
**Study Purpose**	This clinical trial is a feasibility, prospective, interventional, non-randomized multi-center research study evaluating contractility using positive left ventricular (LV) dP/dt max between various LV pacing site(s) in patients indicated for cardiac resynchronization therapy (CRT).
**Background**	CRT has been one of the most important advancements in the past decade for patients with systolic heart failure (HF) and a wide QRS. Several clinical trials have shown improvements in mortality [1, 2], exercise capacity [1], clinical symptoms [2, 3], and quality of life (QOL). However, only approximately 70% of the CRT patients show benefit from the therapy. Multiple reasons for such a low responder rate have been proposed, such as underlying HF severity and etiology, suboptimal device programming, inadequate viable myocardium, lack of baseline dyssynchrony, and non-optimal LV lead position [1-3]. Recent efforts to increase the responder rate is to pace from multiple sites [4]. Multi-site pacing can be achieved either by the introduction of a second LV or right ventricular (RV) lead [5-10] or by additional electrodes on one lead (e.g. Quadripolar system) [11-13]. In a study by Leclercq and coworkers, patients randomized to dual LV pacing had significantly higher LV ejection fraction and smaller LV end-systolic volumes than those randomized to standard BiV (single LV) pacing in the postero-lateral or lateral vein [7]. However, there was no difference in the primary endpoints of QOL and 6 minute hall walk. The use of one-lead with multiple electrodes has recently been investigated in more detail with regard to improving (hemodynamic) response in CRT patients. However, it has been shown that the acute hemodynamic response within one vein is not enhanced by using more spots simultaneously (Multispot) [14]. Recently the importance of a two LV lead system was compared to a Multispot configuration. Multispot pacing alone did not improve the positive dP/dt max when compared to standard BiV pacing, but Multispot pacing using an additional LV lead did increase positive dP/dt max[15].<br><br>The iSPOT study will evaluate three different configurations (BiV, MultiVein and, MultiSpot) and hypothesize that the positive LV dP/dt max achieved by the one-lead multiple electrode configuration (MultiSpot) or two-vein configuration (MultiVein) will be similar to that achieved by the current standard BiV lead configuration (non-inferiority).
**Study Components**	The following components will be used in the study:<br>• Medtronic hand-held pacer<br>• Right atrial, right ventricle and left ventricle leads

Version 3
5 Feb 2014

iSPOT
Summary Clinical Investigation Plan
Medtronic Confidential

1 of 5

	• Left ventricular pressure wires<br>• Blood pressure monitor<br>• Medtronic EP-catheters<br>• Medtronic guiding catheters<br>• CareLink Programmer<br>• Analyzer<br>• Surgical connection cables<br>• Adaptor<br>• Reference lead<br>• Catheter connecting cable, 10 conductor<br>• Connection Box and connectors<br>The Connection Box is investigational. All other products used in the study are commercially released and used within their intended purpose.
**Study Design**	A hemodynamic study will be done prior to an already planned electrophysiological (EP) procedure or CRT-implant. Subject will undergo Magnetic Resonance Imaging (MRI) to locate scar tissue and a venogram will be obtained to identify the target vessels for LV stimulation. The following LV pacing configurations will be evaluated:<br><br>**Biventricular pacing (BiV)**<br>RV lead and one LV lead in three different coronary veins. In total five different pacing settings will be evaluated (figure 1a)<br>**Quadri-ventricular pacing (MultiSpot)**<br>RV lead and 3 "spots" on EP catheter or LV lead placed in lateral vein will be paced simultaneously (figure 1b)<br>**Triventricular pacing (MultiVein)**<br>RV lead, and posterior and anterior LV leads (figure 1c)<br><br>The corresponding positive LV dP/dt will be measured in a repeated way to reduce variability at five different AV delay's with a Millar pressure catheter. Additionally, (non)invasive blood pressure will be measured. Pacing protocol includes synchronized pacing from the RV and LV electrode(s).

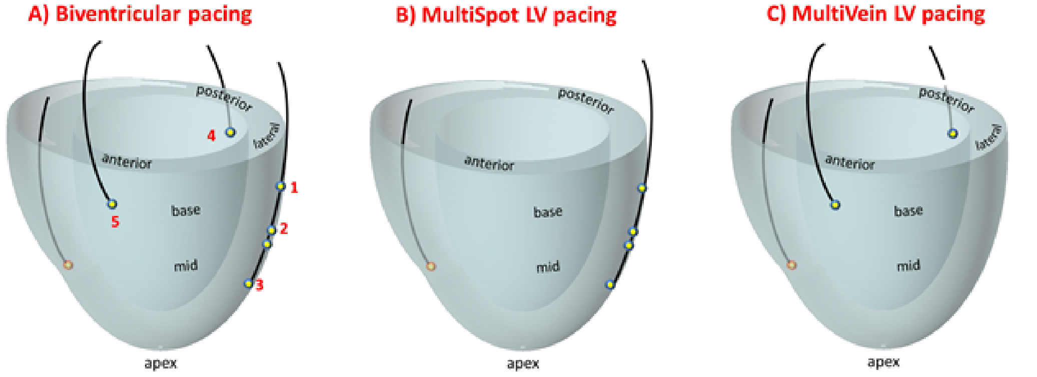

Figure 1: Different ventricular lead positions. 1A) BiV pacing at 5 different settings in three coronary veins. 1= RV – LV Lateral – Proximal (Reference: Standard CRT); 2= RV – LV Lateral – Mid; 3= RV – LV Lateral – Distal; 4=RV – LV Posterior; 5=RV – LV Anterior. 1B) MultiSpot pacing at 3 "spots" in lateral vein simultaneously. 1C) MultiVein pacing in posterior and anterior vein simultaneously

**Study Scope**	The study will be conducted in approximately 86 centers in Europe and Central Asia (ECA) and Middle East and Africa (MEA) in order to meet study enrollment needs in a timely manner. It is anticipated that up to 40 subjects will be enrolled in an enrollment period of one year to evaluate the primary objective.
**Study Objectives**	**Primary objective**<br>Compare the hemodynamic response of a multispot LV pacing configuration to a single spot LV pacing configuration (BiV) in patients undergoing a research study, an EP exploratory procedure or CRT-implant using the contractility parameter positive LV dP/dt max.<br><br>**Secondary Objectives**<br>• Compare the positive LV dP/dt max from the multi-vein LV pacing configuration to the standard single spot LV pacing.<br>• Compare the positive LV dP/dt max from the multi-vein LV pacing configuration to the multispot LV pacing.<br>• Correlate the (non)invasive measures (blood pressure, electrocardiographic mapping and RV/LV EGM timings) obtained during the three pacing configuration's to the positive LV dP/dt max measures obtained.<br>• Evaluate the non-invasive measures ability to identify the pacing configuration with the highest positive LV dP/dt max.<br>• Evaluate the within patient variability in positive LV dP/dt max measures

Inclusion Criteria	Exclusion criteria
• Subject is indicated for CRT or CRT-D device according to current applicable ESC/AHA guidelines<br>• Subject has a left bundle branch block (LBBB) conduction pattern<br>• Subject is in stable sinus rhythm at the time of the EP visit.<br>• Subject receives optimal heart failure oral medical therapy (ACE inhibitor and/or ARB and Beta Blockers).<br>• Subject (or the legal guardian) is willing to sign informed consent form<br>• Subject is 18 years or older or as specified minimal age per local law/regulation	• Subject has permanent atrial fibrillation/ flutter or tachycardia<br>• Subject experienced recent myocardial infarction (MI), within 40 days prior to enrollment.<br>• Subject underwent coronary artery bypass graft (CABG) or valve surgery, within 90 days prior to enrollment<br>• Subject is post heart transplantation, or is actively listed on the transplantation list<br>• Subject is implanted with a left ventricular assist device (LVAD)<br>• Subject has severe renal disease (up to physicians discretion)<br>• Subject is on continuous or uninterrupted infusion (inotropic) therapy for heart failure (≥ 2 stable infusions per week)<br>• Subject has severe aortic stenosis (with a valve area of <1.0 cm2 or significant valve disease expected to be operated within study period)<br>• Subject has complex and uncorrected congenital heart disease<br>• Subject has a mechanical heart valve<br>• Pregnant or breastfeeding women, or women of child bearing potential and who are not on a reliable form of birth control<br>• Subject is enrolled in one or more concurrent studies that would confound the results of this study

References

1. Bristow, M.R., et al., *Cardiac-resynchronization therapy with or without an implantable defibrillator in advanced chronic heart failure.* N Engl J Med, 2004. **350**(21): p. 2140-50.

2. Cleland, J.G., et al., *The effect of cardiac resynchronization on morbidity and mortality in heart failure.* N Engl J Med, 2005. **352**(15): p. 1539-49.

3. Abraham, W.T., et al., *Cardiac resynchronization in chronic heart failure.* N Engl J Med, 2002. **346**(24): p. 1845-53.Anselme, F., et al., *Effect of RV lead(s) site optimization and tri-ventricular pacing in patients undergoing cardiac resynchronization therapy: Results from the METEOR Study.* Europace, 2009. **11 (supplement 2)**.

4. Sanaa, I., et al., *Is there a need for more than one left ventricular lead in some patients?* Europace, 2009. **11 Suppl 5**: p. v29-31.

5. Anselme, F., et al., *Effect of RV lead(s) site optimization and tri-ventricular pacing in patients undergoing cardiac resynchronization therapy: Results from the METEOR Study*. Europace, 2009. **11 (supplement 2)**.

6. Bordachar, P., et al., *Addition of a second LV pacing site in CRT nonresponders rationale and design of the multicenter randomized V(3) trial*. J Card Fail, 2010. **16**(9): p. 709-13.

7. Leclercq, C., et al., *A randomized comparison of triple-site versus dual-site ventricular stimulation in patients with congestive heart failure*. J Am Coll Cardiol, 2008. **51**(15): p. 1455-62.

8. Lenarczyk, R., et al., *Triple-site biventricular pacing in patients undergoing cardiac resynchronization therapy: a feasibility study*. Europace, 2007. **9**(9): p. 762-7.

9. Lenarczyk, R., et al., *Triple-site versus standard cardiac resynchronization therapy study (TRUST CRT): clinical rationale, design, and implementation*. J Cardiovasc Electrophysiol, 2009. **20**(6): p. 658-62.

10. Yoshida, K., et al., *Effect of triangle ventricular pacing on haemodynamics and dyssynchrony in patients with advanced heart failure: a comparison study with conventional bi-ventricular pacing therapy*. Eur Heart J, 2007. **28**(21): p. 2610-9.

11. Bogaard, M.D., et al., *Baseline left ventricular dP/dtmax rather than the acute improvement in dP/dtmax predicts clinical outcome in patients with cardiac resynchronization therapy*. Eur J Heart Fail, 2011. **13**(10): p. 1126-32.

12. Shetty, A.K., et al., *Initial single-center experience of a quadripolar pacing lead for cardiac resynchronization therapy*. Pacing Clin Electrophysiol. **34**(4): p. 484-9.

13. Shetty, A.K., et al., *Quadripolar left ventricular lead implantation through the anchor struts of a mitral valve annuloplasty device*. Europace. **13**(4): p. 590-1.

14. Shetty, A.K., et al., *The Acute Hemodynamic Response to LV Pacing within Individual Branches of the Coronary Sinus using a Quadripolar Lead*. Pacing Clin Electrophysiol, 2011.

15. Shetty, A.K., et al., *Quad-Site Pacing Using a Quadripolar Left Ventricular Pacing Lead*. Pacing Clin Electrophysiol, 2011.

Appendix B: Patient Informed Consent Sample

DESCRIPTION AND PURPOSE

You are invited to take part in a research study called “Left ventricular multispot pacing for cardiac resynchronization therapy (iSPOT)” which is sponsored by Medtronic, Inc.

Your doctor has determined that the pumping ability of your heart is reduced. The two sides of your heart do not beat in synchrony with each other, and as a result, the amount of blood that is pumped out of your heart is reduced and you may have symptoms like shortness of breath or less exercise capacity. Therefore you will receive a Cardiac Resynchronization Therapy (CRT) system to help the heart beat in a more balanced way. The CRT system consists of a CRT device, a right atrial lead (thin wire), a right ventricular lead and a left ventricular lead. Figure 1 shows the system and the location of the leads in the heart. You might undergo an already planned electrophysiologic (EP) procedure prior to the CRT implant. This EP procedure will assess the electrical conduction system of your heart via wires that are temporarily placed in your heart.

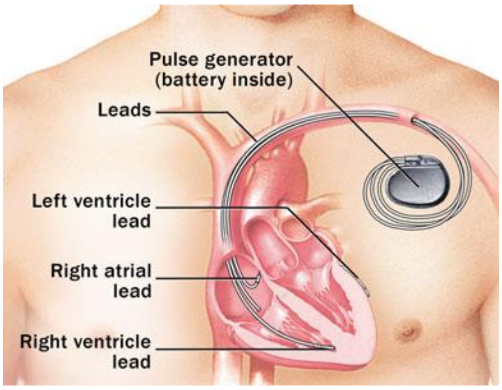

Figure 1: Location of CRT device (pulse generator) and the leads

The response to CRT depends on the position of the leads. Currently, patients who receive a CRT device will have a left ventricular lead implanted which contains one or two electrodes. In this study, a catheter or lead with three electrodes will be placed in the left ventricle during an EP test. The three electrodes give your doctor more programming options to optimize your CRT therapy. In addition, two left ventricular leads will be placed in two different left ventricular veins to stimulate a greater part of your heart.

The purpose of this study is to determine which lead position results in maximal function of the heart. The heart function will be measured by the contractility of the heart and the blood pressure during the EP test that is part of the study.

About 40 patients will take part in this study, which will be conducted in in Europe and Middle East and Africa . This study may help to identify why some patients are unresponsive to CRT. In addition, it will be evaluated whether blood pressure measurements can be used to identify the response to CRT.

Your participation in this study will last between 1 days and 3 months, depending on the time between signing this informed consent and when the EP test will take place, and the moment of hospital discharge after the EP procedure or CRT implant.

As a participant in the study, you have certain responsibilities. You have the responsibility to be truthful regarding your health and medication history. You are expected to return to the study doctor’s office for the study visits. The medical check-ups are a component of the study and important for data

collection. The medical check-ups take place at the screening visit, the MRI visit and at the EP study visit. You should not take part in this study if you will not be available for the study visit or if you wish to participate in another clinical study in the course of this study. Pregnant or breastfeeding women, or women of child bearing potential and who are not on reliable form of birth control may not participate due to the radiation exposure. If you suspect that you have become pregnant while participating in the study, you have to contact the study doctor immediately. You also have the responsibility to report any injuries, hospitalizations, emergency room visits, or other medical visits, symptoms or complaints to the study doctor or study nurse as soon as possible.

PROCEDURES

At the screening visit you may undergo a standard echocardiogram (ultrasound of the heart) and pregnancy test, if applicable, to determine if you meet the study criteria. Your informed consent will be obtained if you are eligible to participate in the study. Your doctor will collect information about your health, your medical history and medications you are taking. Additionally, an electrocardiogram (ECG) and blood samples of up to approximately 15 ml will be collected. This visit is expected to take approximately 30 minutes to one hour and may be combined with the MRI visit and/or EP study visit.

A Magnetic Resonance Imaging (MRI) scan will be performed during the screening visit or during or separate visit, but at the most 3 months before the study visit where the EP test will be done. This MRI scan might be in addition to your standard treatment. However, it might be that your physician already was planning such an evaluation. The MRI image produces cross-sectional images of your heart and will be used to identify heart tissue with impaired blood flow, if present at all. It is a non-invasive procedure which last usually 45 minutes and requires limited movement inside a scanning device. In addition, a special contrast liquid will be injected.

The EP study will take place either on itself, either immediately before the CRT implant or immediately before the already planned EP procedure. During this EP test, in total 3-4 (temporary) leads will be placed: one lead in the atrium, one in the right ventricle and 1 or 2 temporary leads or catheters will be placed at different positions in the veins of the left ventricle. Figure 2 shows the different lead positions in the ventricles. The leads will be connected to the connection box, which will transmit the electrical signals from a generator to the leads. This connection box is investigational and specifically designed to more efficiently perform the EP study. I In addition to the CRT leads, one additional catheter will be placed inside the cavity of the left ventricle to measure the pressure in your heart. You will be given the standard local anesthesia and sedation. If a non-invasive blood pressure monitor is available at the hospital, the sensor of the blood pressure monitor will be placed on your finger to continuously monitor your blood pressure. During the EP test, information from the different leads, the pressure catheter and the blood pressure monitor will be collected. Fluoroscopic images will also be collected during the different implant procedures. The fluoroscopic images are similar to x-rays, which will help to confirm the location of the leads during their implant. Total fluoroscopy time during the EP test study is expected to be approximately 30 minutes. The EP test will last approximately 1-2 hours.

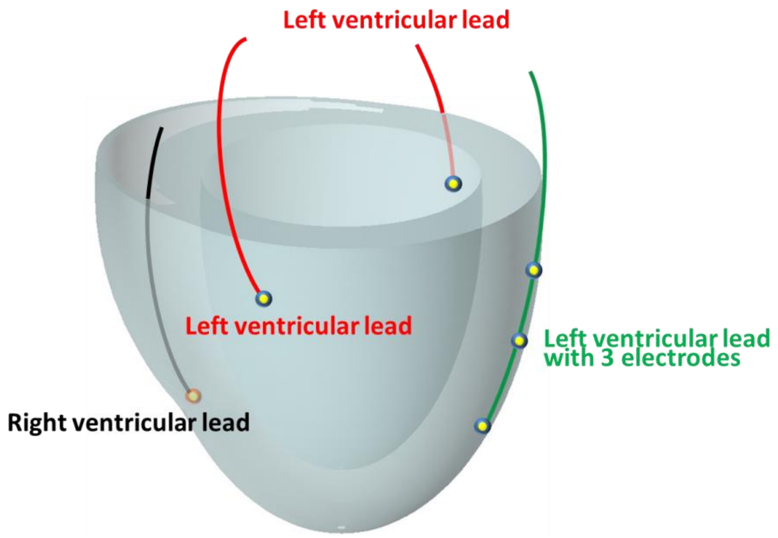

Figure 2: Location of the ventricular leads during EP study. The right ventricular 'black lead' remains during entire EP study. Different positions of left ventricular lead will be assessed: one electrode of the 'green lead', two 'red leads' in two left ventricular veins and one 'green lead' with three electrodes.

After the EP test you may either receive a CRT device and leads according to physician's indication (the physician may use the leads already used in the EP study) **<u>or</u>** a EP procedure will be executed according to physician's indication (the physician most likely uses wires already used in the EP study).

<u>POTENTIAL BENEFITS</u>

Possible benefits in addition to a standard device implant or EP procedure include the following: Electrophysiological information collected from the temporary leads and catheters may be beneficial to your doctor in the management of your heart failure either immediately or during the course of your treatment. It is, however, possible that you may not receive any benefit by participating in the study.

The information from this study can help doctors to understand how to improve the handling and treatment of heart failure, both in your own case and in future patients. Information collected in this study can support the development of new devices and therapies.

<u>RISKS AND INCONVENIENCES</u>

There are possible risks and inconveniences connected with any CRT implant or EP procedure, and these will be discussed separately by your study doctor. These include, but are not limited to: blood clots, infections, bleeding and/or bruising. The additional LV leads and catheters that are used in this study could increase the risk of injury to your veins and/or heart, the formation of blood clots, infection, bleeding or swelling. Additional risks associated with the use of the left ventricular pressure catheter are bruising at the site of blood vessel puncture, hematoma and impaired blood flow to the tissue supplied by the artery. The risks have been minimized through careful study design and manufacture of the leads and catheters. Your study doctor will also help to reduce these risks by using careful implant and EP procedures. Additionally, you will receive blood-thinning medication (heparin) to minimize the risk of blood clot formation. The blood-thinning medication may increase the risk of bleeding and bruising.

The electrical testing during the EP procedure or improper use of the connection box may advertently cause your heart to beat in fast, irregular patterns that could be potentially dangerous to you. If this happens to you, a defibrillation device that will be present could treat these patterns.

A venogram is required for the study. There is a rare chance of developing an allergy due to the X-ray dye which could result in serious injury (such as shock or death). Allergic reaction can usually be treated immediately with medications. The dye may damage your kidney function

Having a MRI scan may mean some added discomfort or inconveniences to you. In particular, you may be bothered by the feelings of claustrophobia and by the loud banging noise during the scan sequences. Temporary hearing loss has been reported from the loud noise. This is why you may be asked to wear earplugs or headphones. Upon standing up after the MRI, your body may feel stiff due to lack of movement and you may also experience mild lightheadedness. During the MRI you may find yourself sweating due to the heat from the MRI machine. You may also find that your body feels warm after the MRI is done. There may be a slight risk that you are allergic to the contrast agent (i.e., gadolinium) used in MRI. Investigators will continuously monitor the procedure and may alter or discontinue the procedure at their discretion for your safety.

The study will extend the total implant or EP procedure time by approximately one hour. This may increase the risk of infection, which can be treated with antibiotics. The protocol procedures will result in an additional 15 minutes of fluoroscopy exposure. Also the time under local anesthesia and sedation will be increased due to prolonged procedure time.

There may be additional discomforts and risks related to the device and/or this study that are not foreseen at this time. However, you will be notified of any significant new findings that develop during the course of the study execution.

ALTERNATIVE PROCEDURES:
If you do not wish to participate in this study, your standard treatment will be continued. Your doctor will discuss the standard treatment with you and the related benefits and risks.

COMPENSATION FOR ILLNESS OR INJURY
If you are physically injured as a result of your participation in this study, reasonable and appropriate medical treatment will be provided to you free of charge by the study sponsor, if such treatment is not already covered by your medical insurance.

Medtronic maintains appropriate clinical trial liability insurance coverage as required under applicable laws and regulations and will comply with applicable local law and custom concerning specific insurance coverage. If required, a Clinical Trial insurance statement/certificate will be provided to your Medical Institution’s Ethics Committee.

COMPENSATION AND ADDITIONAL COSTS
You will not receive any compensation for your participation in this clinical investigation (including follow up).

ROLE OF THE SPONSOR’S REPRESENTATIVE
Sponsor representatives may provide technical support during the EP study. These activities are performed under supervision and responsibility of the investigator and will not bias the data integrity in any way.

USE OF PERSONAL DATA/CONFIDENTIALITY
Your participation in this study is entirely confidential.

While participating in this study, personal information, including medical and health data, will be collected from your medical records. Such information may include on ethnic origins, sexual life or living

habits, or identifying biological samples such as blood samples. These data will be used and processed manually and by computer by Medtronic (meaning the Medtronic, Inc. group of companies). Other designated parties that are involved in the study, including third party data processors, the institution in which you are treated, your physician(s), regulatory authorities and ethics committees, may receive and also be granted access to your personal information in order to comply with legal and regulatory requirements. Your data may be communicated to the above-mentioned parties located in the country in which you are treated and the European Economic Area, India and the United States of America, where the European Directive on Data Protection does not apply.

Your personal data are collected for medical research purposes, to gather information on the device and its performance during and after this study and may be used for additional scientific research, educational purposes and publications as well as for future health studies or for obtaining current or future assessments for approvals for the device.

Your confidential personal information will be anonymized and key-coded, unless it may be impossible to anonymize it, for instance, where your name cannot be removed from the data carrier, such as x-ray, device programming strips and disks, or in order for Medtronic to comply with its reporting obligations. If your data cannot be anonymized, your data will only be accessible by authorized persons (secured access). Study results may be published without disclosing your name or any other identifying characteristics. In all cases, your personal information will be handled at all times in accordance with appropriate confidentiality standards and all applicable data protection and privacy laws.

You are entitled to access the personal information collected about you and to have inaccuracies corrected.

Your personal physician will be informed about your participation in the clinical investigation.

VOLUNTARY PARTICIPATION

Your participation in this study is entirely voluntary. You are free to refuse participation and you are free to discontinue participation in the study at any time without fear of penalty or loss of medical care. In addition, you will be notified of any significant new findings that may develop during the course of the study or the reasons for any amendment to the study protocol, which may relate to your willingness to continue your participation.

Your physician or the sponsor may decide to terminate your participation in the study at any time without your prior consent. If this happens you will be notified and the reasons explained to you.

Medtronic can also suspend or terminate the study at any time without your prior consent. If this happens you will be notified and the reasons will be explained to you. Your physician will continue to provide the appropriate medical treatment.

QUESTIONS

In case of any question about the investigation (e.g. risks, side effects, injury, patient rights, etc.) you can contact:

…………………………………………………………

PATIENT INFORMED CONSENT FORM SIGNATURE SHEET

I have read and understood the patient information of this study and my physician has answered all my questions regarding the study.

I had sufficient time to consider my participation into this study and I am aware that participation into this study is completely voluntary, and I agree to follow the instructions from the investigator.

I realize that I may decide to refuse participation or stop participation at any time without penalty and without affecting the quality of my health care or the relationship with my physician.

I understand and agree that personal information about me will be collected from my medical records, used and processed (manually and by computer) by the manufacturer of a medical device used in my treatment or any other designated party that is involved in the study (e.g. hospital, physician, regulatory authorities, ethics committees).

I understand and agree that representatives from Medtronic, regulatory authorities and the Ethics Committee will be granted direct access to my medical records.

I understand and agree that the physician(s) / hospital will release the relevant personal information about me for the purpose of the clinical investigation.

I understand that I am entitled to access the personal information collected about me and to have inaccuracies corrected.

I have received a copy of the Patient Information and hereby I agree to participate voluntarily in and comply with this study.

I agree to participate in this study and I have consented before the initiation of any study specific procedures.

Patient:

______________________________	______________________	______________________
Name	Signature	Date (dd/MMM/yyyy)
	**! must be written by patient**	**! must be written by patient**

Legal Representative if patient is unable to give consent:

___________________________	____________________	____________________
Name	Signature	Date (dd/ MMM/ yyyy)
	**! must be written by Legal Representative**	**! must be written by Legal Representative**

Investigator or designated person by investigator:
I have conducted the informed consent discussion.

! Only persons officially trained and authorized on the delegated task list are allowed to sign off

___________________________	____________________	____________________
Name	Signature	Date (dd/MMM/yyyy)

If patient, or patient's legally acceptable representative, is unable to read:

I have attended the entire informed consent discussion. I attest that the information in the consent form and any other written information was accurately explained to, and apparently understood by, the patient or the patient's legally acceptable representative. Informed consent was freely given by the patient or the patient's legally acceptable representative.

Impartial Witness:

___________________________	____________________	____________________
Name	Signature	Date (dd/MMM/yyyy
	**! Must be written by impartial witness**	**! Must be written by impartial witness**

Appendix C: References

1. Bristow, M.R., et al., *Cardiac-resynchronization therapy with or without an implantable defibrillator in advanced chronic heart failure.* N Engl J Med, 2004. **350**(21): p. 2140-50.
2. Cleland, J.G., et al., *The effect of cardiac resynchronization on morbidity and mortality in heart failure.* N Engl J Med, 2005. **352**(15): p. 1539-49.
3. Abraham, W.T., et al., *Cardiac resynchronization in chronic heart failure.* N Engl J Med, 2002. **346**(24): p. 1845-53.
4. Fornwalt, B.K., et al., *Agreement is poor among current criteria used to define response to cardiac resynchronization therapy.* Circulation, 2010. **121**(18): p. 1985-91.
5. Butter, C., et al., *Effect of resynchronization therapy stimulation site on the systolic function of heart failure patients.* Circulation, 2001. **104**(25): p. 3026-9.
6. Rossillo, A., et al., *Impact of coronary sinus lead position on biventricular pacing: mortality and echocardiographic evaluation during long-term follow-up.* J Cardiovasc Electrophysiol, 2004. **15**(10): p. 1120-5.
7. Merchant, F.M., et al., *Impact of segmental left ventricle lead position on cardiac resynchronization therapy outcomes.* Heart Rhythm, 2010. **7**(5): p. 639-44.
8. Gold, M.R., et al., *Comparison of stimulation sites within left ventricular veins on the acute hemodynamic effects of cardiac resynchronization therapy.* Heart Rhythm, 2005. **2**(4): p. 376-81.
9. Sanaa, I., et al., *Is there a need for more than one left ventricular lead in some patients?* Europace, 2009. **11 Suppl 5**: p. v29-31.
10. Anselme, F., et al., *Effect of RV lead(s) site optimization and tri-ventricular pacing in patients undergoing cardiac resynchronization therapy: Results from the METEOR Study.* Europace, 2009. **11 (supplement 2)**.
11. Bordachar, P., et al., *Addition of a second LV pacing site in CRT nonresponders rationale and design of the multicenter randomized V(3) trial.* J Card Fail, 2010. **16**(9): p. 709-13.
12. Leclercq, C., et al., *A randomized comparison of triple-site versus dual-site ventricular stimulation in patients with congestive heart failure.* J Am Coll Cardiol, 2008. **51**(15): p. 1455-62.
13. Lenarczyk, R., et al., *Triple-site biventricular pacing in patients undergoing cardiac resynchronization therapy: a feasibility study.* Europace, 2007. **9**(9): p. 762-7.
14. Lenarczyk, R., et al., *Triple-site versus standard cardiac resynchronization therapy study (TRUST CRT): clinical rationale, design, and implementation.* J Cardiovasc Electrophysiol, 2009. **20**(6): p. 658-62.
15. Yoshida, K., et al., *Effect of triangle ventricular pacing on haemodynamics and dyssynchrony in patients with advanced heart failure: a comparison study with conventional bi-ventricular pacing therapy.* Eur Heart J, 2007. **28**(21): p. 2610-9.
16. Bogaard, M.D., et al., *Baseline left ventricular dP/dtmax rather than the acute improvement in dP/dtmax predicts clinical outcome in patients with cardiac resynchronization therapy.* Eur J Heart Fail, 2011. **13**(10): p. 1126-32.
17. Shetty, A.K., et al., *Initial single-center experience of a quadripolar pacing lead for cardiac resynchronization therapy.* Pacing Clin Electrophysiol. **34**(4): p. 484-9.
18. Shetty, A.K., et al., *Quadripolar left ventricular lead implantation through the anchor struts of a mitral valve annuloplasty device.* Europace. **13**(4): p. 590-1.

19. Forleo, G.B., et al., *Left ventricular pacing with a new quadripolar transvenous lead for CRT: early results of a prospective comparison with conventional implant outcomes.* Heart Rhythm, 2011. **8**(1): p. 31-7.
20. Thibault, B., et al., *Pacing electrode selection in a quadripolar left heart lead determines presence or absence of phrenic nerve stimulation.* Europace. **12**(5): p. 751-3.
21. Ellenbogen, K.A. and J. Kron, *Cardiac resynchronization therapy: location matters.* J Am Coll Cardiol, 2011. **58**(5): p. 491-2.
22. Singh, J.P., et al., *Left ventricular lead position and clinical outcome in the multicenter automatic defibrillator implantation trial-cardiac resynchronization therapy (MADIT-CRT) trial.* Circulation. **123**(11): p. 1159-66.
23. Shetty, A.K., et al., *The Acute Hemodynamic Response to LV Pacing within Individual Branches of the Coronary Sinus using a Quadripolar Lead.* Pacing Clin Electrophysiol, 2011.
24. Shetty, A.K., et al., *Quad-Site Pacing Using a Quadripolar Left Ventricular Pacing Lead.* Pacing Clin Electrophysiol, 2011.
25. Duckett, S.G., et al., *Invasive acute hemodynamic response to guide left ventricular lead implantation predicts chronic remodeling in patients undergoing cardiac resynchronization therapy.* J Am Coll Cardiol, 2011. **58**(11): p. 1128-36.
26. Butter, C., et al., *Cardiac resynchronization therapy optimization by finger plethysmography.* Heart Rhythm, 2004. **1**(5): p. 568-75.
27. Whinnett, Z.I., et al., *Determination of optimal atrioventricular delay for cardiac resynchronization therapy using acute non-invasive blood pressure.* Europace, 2006. **8**(5): p. 358-66.
28. Whinnett, Z.I., et al., *Haemodynamic effects of changes in atrioventricular and interventricular delay in cardiac resynchronisation therapy show a consistent pattern: analysis of shape, magnitude and relative importance of atrioventricular and interventricular delay.* Heart, 2006. **92**(11): p. 1628-34.
29. Bocchiardo, M., et al., *Resynchronization therapy optimization by intracardiac impedance.* Europace, 2010. **12**(11): p. 1589-95.
30. Turcott, R.G., et al., *Measurement precision in the optimization of cardiac resynchronization therapy.* Circ Heart Fail. **3**(3): p. 395-404.
31. Jia, P., et al., *Electrocardiographic imaging of cardiac resynchronization therapy in heart failure: observation of variable electrophysiologic responses.* Heart Rhythm, 2006. **3**(3): p. 296-310.
32. Tysler, M., et al., *Noninvasive assessment of local myocardium repolarization changes using high resolution surface ECG mapping.* Physiol Res, 2007. **56 Suppl 1**: p. S133-41.
33. Hsing, J.M., et al., *Paced Left Ventricular QRS Width and ECG Parameters Predict Outcomes After Cardiac Resynchronization Therapy: PROSPECT-ECG Sub-Study.* Circ Arrhythm Electrophysiol, 2011.
34. Clementy, N., et al., *Successful 'quadrangular' pacing in a non-responder patient to cardiac resynchronization therapy.* Eur Heart J, 2011. **32**(17): p. 2215.
